# A NIR-Ⅱ-Immunostimulatory nanoplatform rewires immunometabolism to unleash STING-driven antitumor immunity

**DOI:** 10.1186/s12951-026-04162-2

**Published:** 2026-03-15

**Authors:** Xun Yang, Xuefeng Chen, Minhao Chen, Simei Yang, Ya Wu, Hongye Liao, Tong Xia, Gaoyang Shen, Changzhen Sun, Li Liu

**Affiliations:** 1 Skin Structure and Function Key Laboratory of Luzhou, Department of Dermatology, The Affiliated Hospital, Southwest Medical University, Sichuan Province 646000 Luzhou, China; 2 Drug Research Center of Integrated Traditional Chinese and Western Medicine, The Affiliated Traditional Chinese Medicine Hospital, Southwest Medical University, Sichuan Province 646000 Luzhou, China; 3 Department of Vascular Surgery, The Affiliated Hospital, Southwest Medical University, Sichuan Province 646000 Luzhou, China; 4 Luzhou Key Laboratory of Research and Development of Medical Institution Preparations and Large-scale Health Products, The Affiliated Traditional Chinese Medicine Hospital, Southwest Medical University, Sichuan Province 646000 Luzhou, China

**Keywords:** NIR-Ⅱ imaging, Photothermal therapy, STING pathway, Pyroptosis, Immunometabolic reprogramming

## Abstract

**Background:**

Melanoma represents a highly aggressive and immunotherapy-resistant malignancy with limited immunotherapy efficacy, underscoring the urgent need for novel treatment strategies that integrate precise diagnosis and potent immunomodulation. The combination of photothermal therapy (PTT) and STING pathway activation has emerged as a promising approach to potentiate antitumor immunity. Nevertheless, it remains challenging to integrate real-time deep-tissue imaging with spatiotemporally synchronized immunostimulation within a single nanoplatform, especially for the effective treatment of advanced melanoma.

**Results:**

Herein, we report a mitochondria-targeted nanotheranostic agent (IRM) constructed through molecular co-assembly of a STING agonist (MSA-2) and a lab-synthesized NIR-Ⅱ fluorophore (IR-817). This nanoplatform enables simultaneous NIR-Ⅱ fluorescence imaging and high-efficiency photothermal conversion (η = 52.79%). More importantly, it ensures efficient, on-demand drug action through spatiotemporally controlled delivery. Under 808 nm laser irradiation, IRM induced localized hyperthermia that provoked pyroptosis and immunogenic cell death (ICD) in primary melanoma tumors. Concurrently, the photothermal stimulus promoted the rapid release of MSA-2, which synergistically activated the STING pathway in dendritic cells (DCs). This event drove immunometabolic reprogramming of the tumor microenvironment, elicited a robust systemic cytotoxic T-cell response, and effectively reversed the immunosuppressive state. This cascade of biological events ultimately led to significant inhibition of distant tumors, demonstrating a robust abscopal effect. Crucially, this therapeutic effect was strictly STING-dependent: in *STING*-KO mouse models, the suppression of distant tumors was completely abolished following the same treatment. These complementary experimental outcomes directly confirm the indispensable synergy between PTT and STING pathway activation, which together constitute the core mechanism underlying the induction of systemic antitumor immunity by the IRM nanoplatform.

**Conclusions:**

Our study illustrates that the IRM nanoplatform effectively merges multimodal imaging with immunometabolic modulation, establishing a durable and systemic antitumor immunity. This work offers a translatable strategy for combinational photo-immunotherapy against advanced melanoma.

**Graphical Abstract:**

**Figure:**
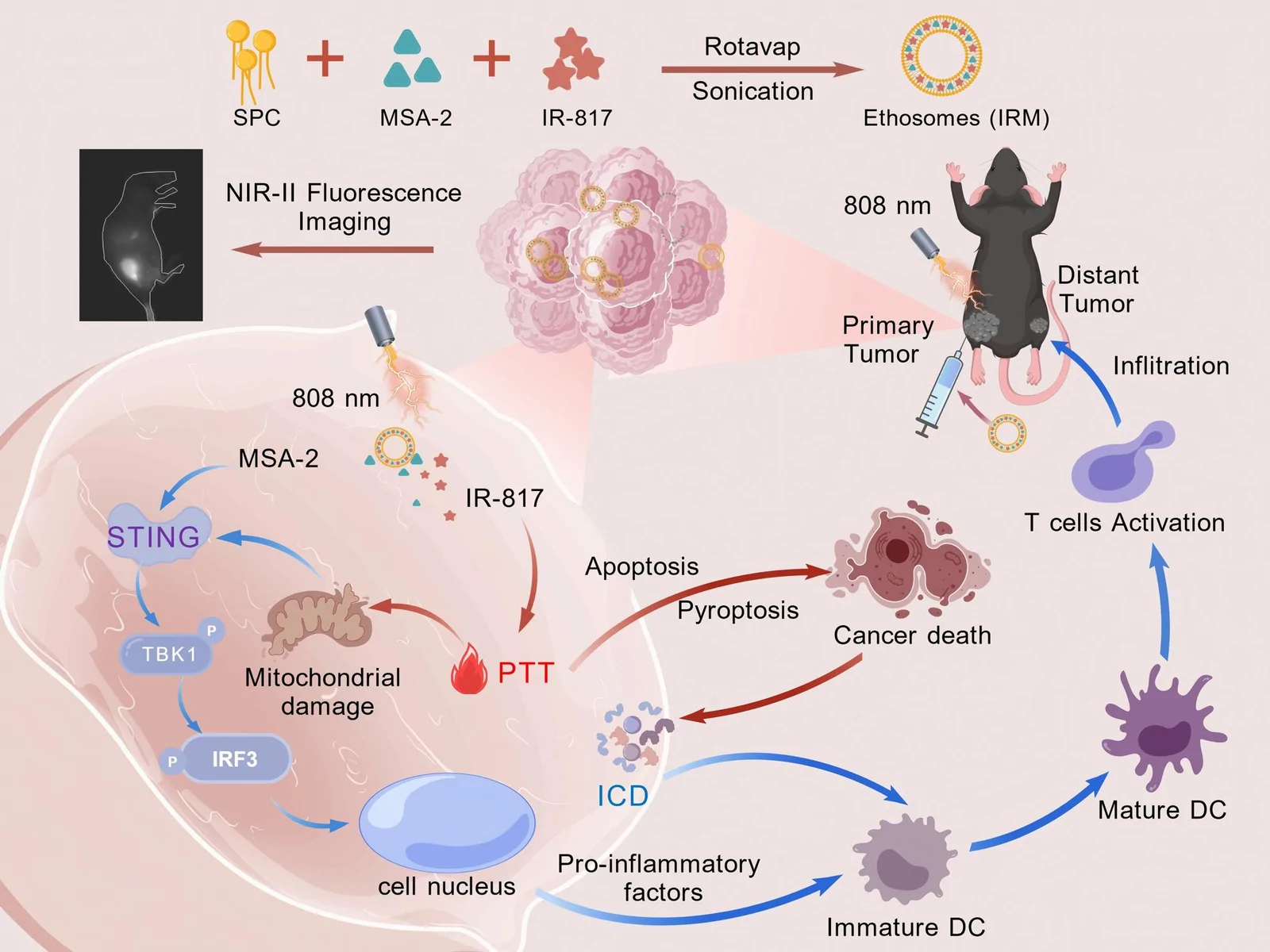

**Supplementary Information:**

The online version contains supplementary material available at 10.1186/s12951-026-04162-2.

## Introduction

Malignant melanoma (MM), although comprising only 5%-10% of all cutaneous malignancies, accounts for more than 75% of skin cancer-associated mortality, representing the most lethal variant of skin cancer [1, 2]. A hallmark of this tumor is its immunosuppressive microenvironment, which restricts immune cell infiltration and thereby impairs the efficacy of antitumor therapies [3, 4]. Currently available immune checkpoint inhibitors (ICIs) and chemotherapy are limited by low response rates [5] and systemic toxicity, respectively, making the treatment of advanced distantly metastatic melanoma particularly challenging [6]. Therefore, the development of novel strategies that can directly eliminate tumor cells, activate antitumor immunity, and improve therapeutic efficacy is an urgent research priority.

IR-817, a heptamethine cyanine dye synthesized by our research group, has been preliminarily confirmed to exhibit fluorescence emission in the first near-infrared window (NIR-I, 700–900 nm). Additionally, it selectively accumulates in the mitochondria of melanoma cells via organic anion-transporting polypeptides (OATPs) and induces apoptosis through activation of the Caspase cascade [7]. Our recent study revealed that IR-817 exhibits dual functionality, demonstrating a temperature increase (ΔT) of 15.7 °C under 808 nm laser irradiation [8]. and as a fluorescence probe for imaging in the second near‑infrared window (NIR‑Ⅱ, 900–1700 nm). Compared with the NIR-I, NIR-Ⅱ fluorescence imaging offers superior spatiotemporal resolution and deeper tissue penetration, making it particularly suitable for tumor imaging [9].

Photothermal therapy (PTT) utilizes photothermal conversion agents to generate localized hyperthermia upon NIR irradiation, enabling selective tumor ablation [10]. Accumulating evidence indicates that PTT-induced thermal shock or lysosomal damage activates the NLRP3 inflammasome, which recruits and activates Caspase-1 to cleave GSDMD into pore‑forming N‑terminal fragment (GSDMD-N), triggering pyroptosis while promoting the maturation and release of IL-1β [11]. Moreover, PTT induces immunogenic cell death (ICD), releasing damage-associated molecular patterns (DAMPs), including calreticulin (CRT) and high-mobility group box 1 (HMGB1) [10]. These DAMPs subsequently facilitate dendritic cell (DC) maturation and enhance antigen presentation, thereby augmenting antitumor immunity [12, 13].

The stimulator of interferon genes (STING) pathway is crucial for cancer immune surveillance [14–16]. Its activation promotes dendritic cell (DC) maturation [15, 17, 18] and enhances the maintenance of stem-like CD8^+^ T cells [19, 20]. Mechanistically, STING activation upregulates key signaling molecules, including *p*TBK1 and *p*IRF3 [21–23], driving the secretion of type I interferons and other inflammatory cytokines. This cascade enhances DC cross-presentation, activates tumor-specific T cells, and remodels the tumor microenvironment (TME) [24, 25]. Studies have demonstrated that localized PTT can induce both immunogenic signals and pyroptosis. Specifically, PTT-generated immunogenic signals promote the activation of the cyclic GMP-AMP synthase cGAS-STING pathway to further amplify immune responses [26]. On the other hand, localized hyperthermia triggers pyroptosis via the GSDMD pathway, which in turn elicits antitumor immunity [27–29]. Consequently, combining PTT not only directly ablates tumor cells but also enhances immune responses by promoting DC maturation and increasing the frequency of tumor-infiltrating T cells [30, 31]. Building on this, the combination of STING agonists with other therapeutic approaches has gained increasing attention. MSA-2, a novel small-molecule STING agonist that can be efficiently taken up by the acidic TME, significantly enhances cytokine expression and activates the STING pathway. However, it faces limitations such as pronounced off-target effects, a short in vivo duration of action (approximately 2–4 h), and considerable systemic toxicity [32, 33]. To address these issues, nanodelivery strategies have been widely employed to optimize the pharmacokinetic profile of MSA-2. including covalent conjugation and nanoassembly to improve its lymph node targeting and retention [34], as well as sequential release systems to extend its action time and enhance combination efficacy [33].

PTT-induced pyroptosis is recognized for its rapid and potent effects [11], yet concerns regarding systemic inflammation highlight the need for precise activation strategies. This necessitates accurate tumor targeting to minimize off-target damage, prompting the adoption of NIR-Ⅱ fluorescence imaging-guided PTT as a strategic solution [27, 30, 35]. Such a combination not only leverages the deep tissue penetration and high resolution of NIR-Ⅱ imaging to achieve precise tumor localization but also enables spatiotemporally controlled PTT to induce pyroptosis specifically in tumor cells, which is consistent with the core design concept of nanomedicine for targeted pyroptosis induction.

Ethosomes represent an advanced nanodelivery system with remarkable advantages. Their high ethanol content (20%-50%) significantly enhances biomembrane permeability, enabling deep tissue penetration and drug delivery. This system exhibits efficient loading capacity for diverse drug molecules, including hydrophilic, hydrophobic, and macromolecular agents, while maintaining excellent stability at ambient temperature [36, 37].Previous studies have utilized ethosomes as a drug delivery platform for melanoma therapy, which have been shown to improve drug solubility, regulate drug release, prolong drug retention, and ultimately enhance therapeutic efficacy [38–40].

**Scheme 1:**
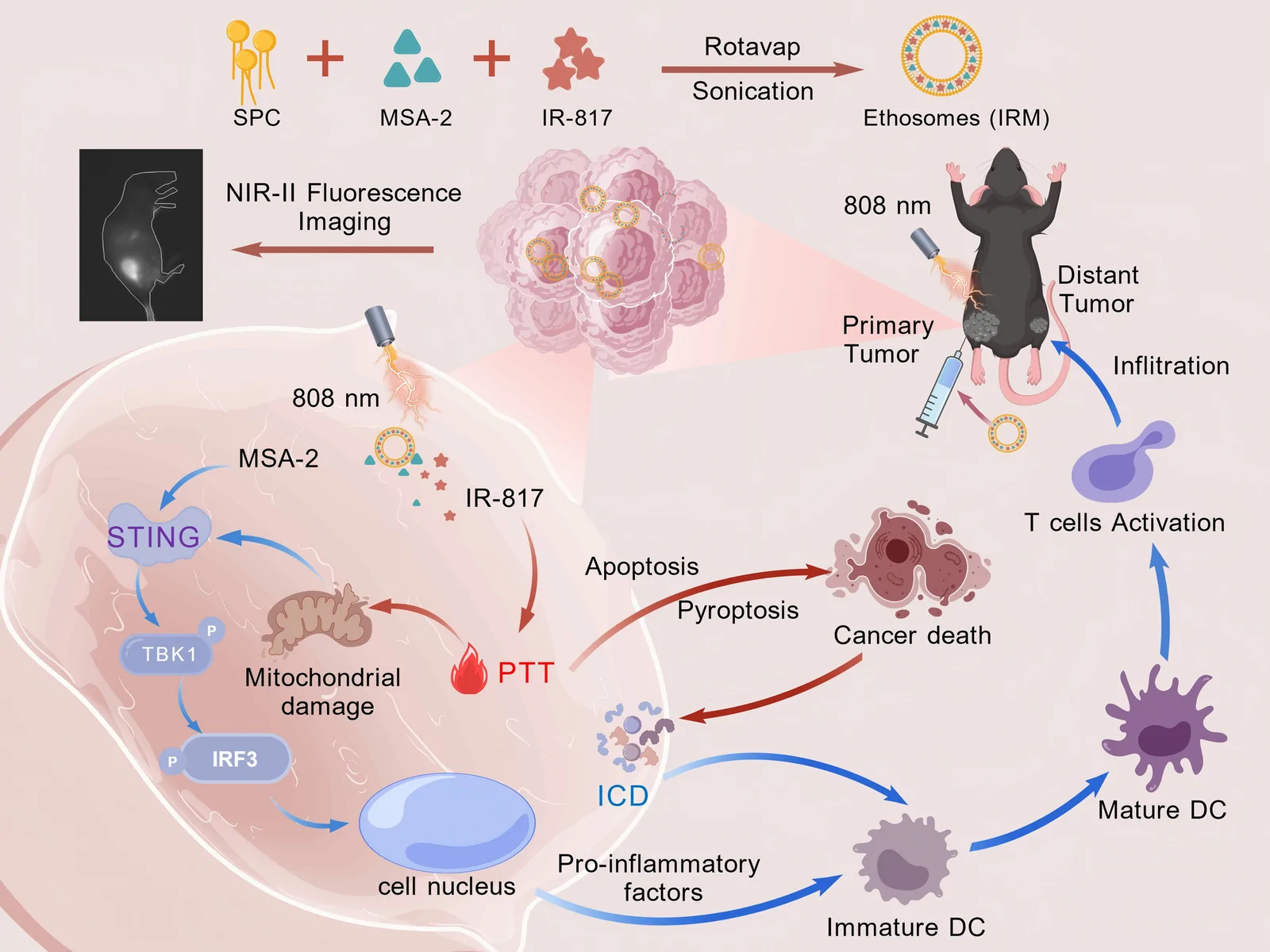
NIR-II Fluorescence Imaging-Guided Synergistic Photothermal and STING Agonist Therapy for Local and Systemic Antitumor Immunity

Based on the aforementioned advances, this study developed an ethosomal nanoplatform co-delivering MSA-2 and IR-817, which possesses both photothermal therapy (PTT) and NIR‑II imaging capabilities. Integrating triple functions of NIR-Ⅱ imaging, PTT, and STING immunotherapy (Scheme.1): NIR-Ⅱ fluorescence navigation enabled precise tumor margin delineation and real-time monitoring of treatment responses; PTT not only directly ablates primary tumors but also provides antigen sources through pyroptosis and ICD effects; while MSA-2-mediated STING activation overcomes immunosuppressive barriers in the TME, establishing durable antitumor immune memory. This “visualization-local therapy-systemic regulation” strategy demonstrated synergistic efficacy in both primary tumor regression and distant tumor suppression in B16 subcutaneous dual-tumor models, offering a novel paradigm for precision theranostics of melanoma.

## Materials and methods

### Materials

MSA-2(2’,3’-Di-O-(2,4-dinitrophenyl)-inosine-5’-monophosphate) was purchased from Sic huan Weikeqi Biological Technology Co.Ltd. (Sic huan, China), Soybean phospholipid was obtained from Shanghai Yuanye Bio-Technology Co.Ltd. (Shanghai, China), Dulbecco’s Modified Eagle Medium, RPMI 1640 and fetal bovine serum (FBS) were purchased from BasalMedia (Shanghai, China), trypsin-EDTA, penicillin-streptomycin were supplied by Solarbio (Shanghai, China). 3-(4,5-diMethylthialzol-2-yl)-2,5-diphe-nyltetrazolium bromide (MTT) and Hoechst33342 were purchased from YuanYe Bio-Technology (Shanghai, China). The universal SP kit (mouse/rabbit streptavidin-biotin detection system), DAB kit and Phosphate-Buffered Saline (PBS) buffer (pH 7.4) were purchased from Zsbio (Beijing, China). All-purpose Powerful Antigen Retrieval Solution and Calcein AM/PI Live/Dead Assay kit and the Immunofluorescence Staining Kit were purchased from Beyotime Institute of Biotechnology (Shanghai, China). Protein Loading Buffer, Multicolor Prestained Protein Ladder and 10% SDS-PAGE were from EpiZyme (Shanghai, China). Near-infrared dye IR-817 was synthesized following our previously reported protocol. PE-anti-mouse CD80, APC-anti-mouse CD86, FITC-anti-mouse CD11c, and their corresponding isotype control antibodies were purchased from LinkBio (Chengdu, China), an authorized distributor of BioLegend. Antibody against Ki-67 was purchased from Cell Signaling Technology (CST, Beverly, MA, USA). Antibodies against STING, TBK1 and phosphorylated TBK1, IRF3 and phosphorylated IRF3 were purchased from Beyotime Biotechnology Co.Ltd. (Shanghai, China). Antibodies against phosphorylated STING (TMEM173) (Ser366), FOXP3 and CD206 were purchased from Immunoway (Shanghai, China). Antibody against Caspase1 was purchased from Solarbio (Beijing, China). Antibody against NLRP3 was purchased from MCE (USA). Antibody against GSDMD was purchased from Huabio (Hangzhou, China). Antibody against β-actin was purchased from Zen-bio (Chengdu, China).

### Synthesis and characterization of IRM

Nanoplatform co-loaded with MSA-2 and IR-817 (hereinafter referred to as IRM), were prepared using the thin-film hydration method. Briefly, 10 mg MSA-2, 25 mg IR-817 (hereinafter referred to as IR), and 450 mg soybean phospholipids were dissolved in 30 mL of a chloroform/methanol mixture (v/v = 2:1). A thin lipid film was formed by rotary evaporation at 35 °C under vacuum, followed by further drying to completely remove residual solvents. The film was hydrated with 44 mL of 20% ethanol aqueous solution under agitation to form a homogeneous suspension, which was then sonicated for 20 min. The prepared samples were stored at 4 °C. Finally, the suspension was filtered through a 220 nm microporous membrane to obtain a uniform nanoparticle solution. The nanoparticles were characterized by UV-Vis spectrophotometry (UV-1800, Shimadzu, Japan), dynamic light scattering (Malvern Panalytical, UK), and transmission electron microscopy (TEM). Following the same procedure, Nanoplatform loaded solely with IR (hereinafter referred to as IRE), Nanoplatform loaded solely with MSA-2 (hereinafter referred to as MES) were prepared separately.

### Determination of encapsulation efficiency and drug loading capacity of IRM

The encapsulation efficiency (EE%) and drug loading capacity (DLC%) of IRM were evaluated. The UV absorbance peaks at 800 nm and 327 nm were measured using a NanoDrop One spectrophotometer (Thermo Fisher Scientific, USA). The absorbance values were then applied to the standard curves of IR and MSA-2 to determine drug concentration and mass. EE% and DLC% were calculated according to Eqs. (1) and (2):

1 $$\mathrm{EE}\% = (\mathrm{W}_a/\mathrm{W}_\mathrm{Initial}) \times 100\%$$

2 $$\mathrm{DLC}\% = (\mathrm{W}_\mathrm{a}/\mathrm{W}_\mathrm{all}) \times 100\% $$

where W_a_ represents the calculated drug mass, W_Initial_ denotes the initially added drug mass, and W_all_ refers to the total calculated mass of each drug in the nanoparticles.

### Photothermal effect of IRM

Solutions containing varying concentrations of IRM were subjected to NIR laser irradiation (808 nm, 1.0 W/cm^2^) for 5 min. Temperature variations were monitored in real-time using an infrared thermal imaging system (FLIR E50) during exposure to different laser power intensities. The heating-cooling profiles of IRM were recorded over three complete cycles, from which temperature change curves were plotted and the photothermal conversion efficiency (η) was calculated [39–41].

### In vitro release experiment

The in vitro release behavior of the nanomedicine was evaluated using the dynamic dialysis method. In brief, an accurately measured volume of the nanoformulation (1 mL of the stock solution) was placed into a pretreated dialysis bag, which was then immersed in 50 mL PBS at pH 7.4 or pH 6.5. The entire system was incubated in a thermostatic shaker at 37 °C with agitation at 100–150 rpm to simulate physiological conditions. After irradiating the release system with 808 nm laser (1.0 W/cm², 10 min), samples of the external medium (2 mL) were withdrawn at predetermined time points (0.5, 1, 2, 4, 8, 12, 24, 48, 72 h.), with immediate replenishment by an equal volume of fresh, prewarmed medium. The collected samples were appropriately processed, and the drug concentration was determined by UV-Vis spectrophotometry to calculate the cumulative release percentage. All experiments were performed independently in triplicate.

### Evaluation of NIR-Ⅱ fluorescence imaging for IRM

Fluorescence emission spectra of IRM were recorded at 10 µg/mL (600–1000 nm) and 5 µg/mL (800–1600 nm) in aqueous solution. NIR-Ⅱ fluorescence images were acquired for IR, IRE, and IRM at varying concentrations in different matrices using a NIR-Ⅱ fluorescence imaging system. In vivo NIR-Ⅱ fluorescence imaging was performed on tumor-bearing mice post intravenous injection (IR dose: 2 mg/kg) with time-lapse recording. All measurements were conducted under standardized conditions.

### Cell culture and uptake studies

A375 and B16 cells (ATCC) were maintained in RPMI 1640 medium (Gibco) supplemented with 10% FBS and 1% penicillin/streptomycin at 37 °C with 5% CO_2_ (≤ 5 passages). For cellular uptake, A375 and B16 cells (2 × 10^5^ cells/well) were seeded in 6-well plates overnight. IR and IRM (20 µg/mL) were added to wells with varying incubation times. Fluorescence images were acquired using a fluorescence microscope (Leica, Germany). All imaging conditions were kept consistent across groups.

### Photothermal therapy assessment

A375 and B16 cells were treated with varying concentrations of IRM for 24 h. For the laser-treated group, cells were irradiated (808 nm, 1.0 W/cm^2^, 5 min) at 12 h post-treatment, followed by continued incubation. MTT assay was performed by adding 20 µL of 5 mg/mL MTT solution per well for 2 h incubation. The formazan crystals were dissolved in 150 µL DMSO for 15 min. Absorbance at 570 nm was measured using a Spectra MAX M5 microplate reader (Molecular Devices) to calculate cell viability. All experiments were performed in triplicate.

### Cytotoxicity evaluation

BJ human foreskin fibroblasts and COS-1 kidney cells were treated with MES, IRE or IRM for 24 h, followed by MTT assay (OD 570) to determine viability.

### Cell viability assessment by Calcein-AM/PI staining

A375 and B16 cells (2 × 10^5^ cells/well) were seeded in 6-well plates overnight and treated with 2 µg/mL IR, IRE and IRM. The laser-treated group received NIR irradiation (808 nm, 1.0 W/cm^2^, 5 min) at 12 h post-treatment, followed by additional 12 h incubation. Cells were then washed with PBS and co-stained with Calcein-AM (for live cells) and Propidium Iodide (PI, for dead cells). Fluorescence imaging was performed using a fluorescence microscope (Leica, Germany). Calcein-AM (green) and PI (red) fluorescence were acquired using appropriate filter sets.

### Western blot analysis

Cells were harvested after treatment and lysed in RIPA buffer containing 1% PMSF. Total protein was extracted following sonication and quantified using a BCA protein assay kit. Protein samples were denatured in loading buffer and separated by 8%-12% SDS-PAGE, then transferred to PVDF membranes. Membranes were blocked with rapid blocking solution for 15 min at Room Temperature (RT) and incubated with primary antibodies overnight at 4 °C. After washing, membranes were probed with HRP-conjugated secondary antibodies (anti-rabbit or anti-mouse, 1.5 h, RT). Protein bands were visualized using ECL reagent on a UVP ChemStudio PLUS system (Analytikjena) and quantified with ImageJ software.

### Flow cytometric analysis of BMDC maturation

Bone marrow-derived dendritic cells (BMDCs) were isolated from femurs and tibias of 6-8-week-old female C57BL/6J mice euthanized by cervical dislocation. Bone marrow was flushed with RPMI-1640 medium, and erythrocytes were lysed using RBC lysis buffer. Cells were cultured in DC-specific medium (RPMI-1640 containing 20 ng/mL GM-CSF and 10 ng/mL IL-4) for 7 days with medium changes on day 3 and 5. Immature BMDCs (day 7) were adjusted to 3 × 10⁶ cells/mL and divided into five experimental groups (Control, MES, IRE + L, IRM, IRM + L) plus isotype controls. After 2 h plating, treatments were added for 12–14 h, followed by laser irradiation (808 nm, 1.0 W/cm², 5 min) where indicated. Cells were stained with anti-CD11c-FITC, anti-CD80-PE, and anti-CD86-APC (5 µL each) for 30 min at RT, fixed with 4% paraformaldehyde (PFA), and analyzed by flow cytometry. Data were processed using GraphPad Prism.

### Immunofluorescence assay

Cell preparation and fixation: Briefly, cells were seeded on glass coverslips placed in a 24-well plate and allowed to adhere overnight. Following the respective treatments, the cells were washed gently with PBS and fixed with 4% PFA for 15–20 min at RT.

Permeabilization and blocking: The fixed cells were then permeabilized with 0.1% Triton X-100 in PBS for 10 min to allow antibody access to intracellular targets. Afterward, the cells were incubated in a blocking solution (5% bovine serum albumin (BSA) in PBS for 1 h at RT to prevent nonspecific antibody binding.

Antibody incubation: The cells were incubated overnight at 4 °C with the primary antibodies. The following day, after extensive washing with PBS, the cells were incubated with appropriate fluorophore-conjugated secondary antibodies (Alexa Fluor 488 or 594) for 1 h at RT in the dark. Nuclei were counterstained with Hochest 33342.

Image acquisition: The coverslips were mounted onto glass slides using an anti-fade mounting medium. Fluorescent images were captured using a confocal laser scanning microscope under consistent exposure settings for each channel.

### Establishment of subcutaneous dual-tumor model and treatment protocol

The animal study protocol was approved by the Ethics Committee of Southwest Medical University (protocol code SWMU20250061). Female C57BL/6J mice (4–6 weeks old) were subcutaneously inoculated with 4 × 10⁵ B16 cells in the right dorsal flank to establish primary tumors. distant tumors were generated by implanting an equal number of cells in the left dorsal flank 7 days later. When the primary tumors reached 100–200 mm³, the mice were randomly divided into six groups (*n* = 6): Vehicle, Vehicle + L, IRE, IRE + L, IRM, and IRM + L. Drugs (2 mg/kg) were intratumorally injected into primary tumors, followed by NIR laser irradiation (808 nm, 1.0 W/cm², 5 min) at 14 h post-injection. Treatments were administered every 3 days for three cycles. Tumor dimensions and body weight were monitored every 3 days. All animal procedures were approved by the Institutional Animal Care and Use Committee of Southwest Medical University. Tumor volume was calculated using the formula: V = (length × width²)/2.

### Hematoxylin and Eosin (H&E) staining

The collected tissues were fixed with 4% PFA over night, then dehydrated with ethanol, embedded in paraffin and cut into 4 μm-thick sections, and eventually stained with H&E, sealed with resin, and observed using a microscope (Nikon, NiE, Japan).

### Immunohistochemistry

The paraffin-embedded tissues were cut into 4 μm-thick sections in the slides, which were heated in antigenic repair solution at 95 ℃ for 20 min. The sections were soaked in 0.25% potassium permanganate for 6 min and then in 2% oxalic acid for 4 min to remove melanin. After using the universal SP kit (mouse/rabbit streptavidin-biotin detection system) according to the manufacturer’s instructions, antibodies were individually incubated in tissue slides at 37 ℃ for 1 h. Subsequently, which incubated with HRP-conjugated goat anti-rabbit/mouse IgG for 15 min at 37 ℃. In the end, horseradish peroxidase activity was detected using a DAB kit.

### In vivo biosafety evaluation

On day 9 post-treatment, blood samples were collected via orbital bleeding and centrifuged to obtain serum for biochemical analysis. Upon reaching experimental endpoints, major organs (liver, heart, spleen, lung, and kidney) were harvested for histological examination by H&E staining. Serum biochemical parameters included liver/kidney function markers and inflammatory cytokines.

### Statistical analysis

Data analyses were conducted using the GraphPad Prism 10 software. For variance analysis, One-way analysis of variance (ANOVA) with Tukey’s post hoc test was used. p values of < 0.05 were considered significant. *p < 0.05, **p < 0.01, ***p < 0.001, and ****P < 0.0001.

## Results and discussion

### Synthesis and characterization of IRM

**Fig. 1:**
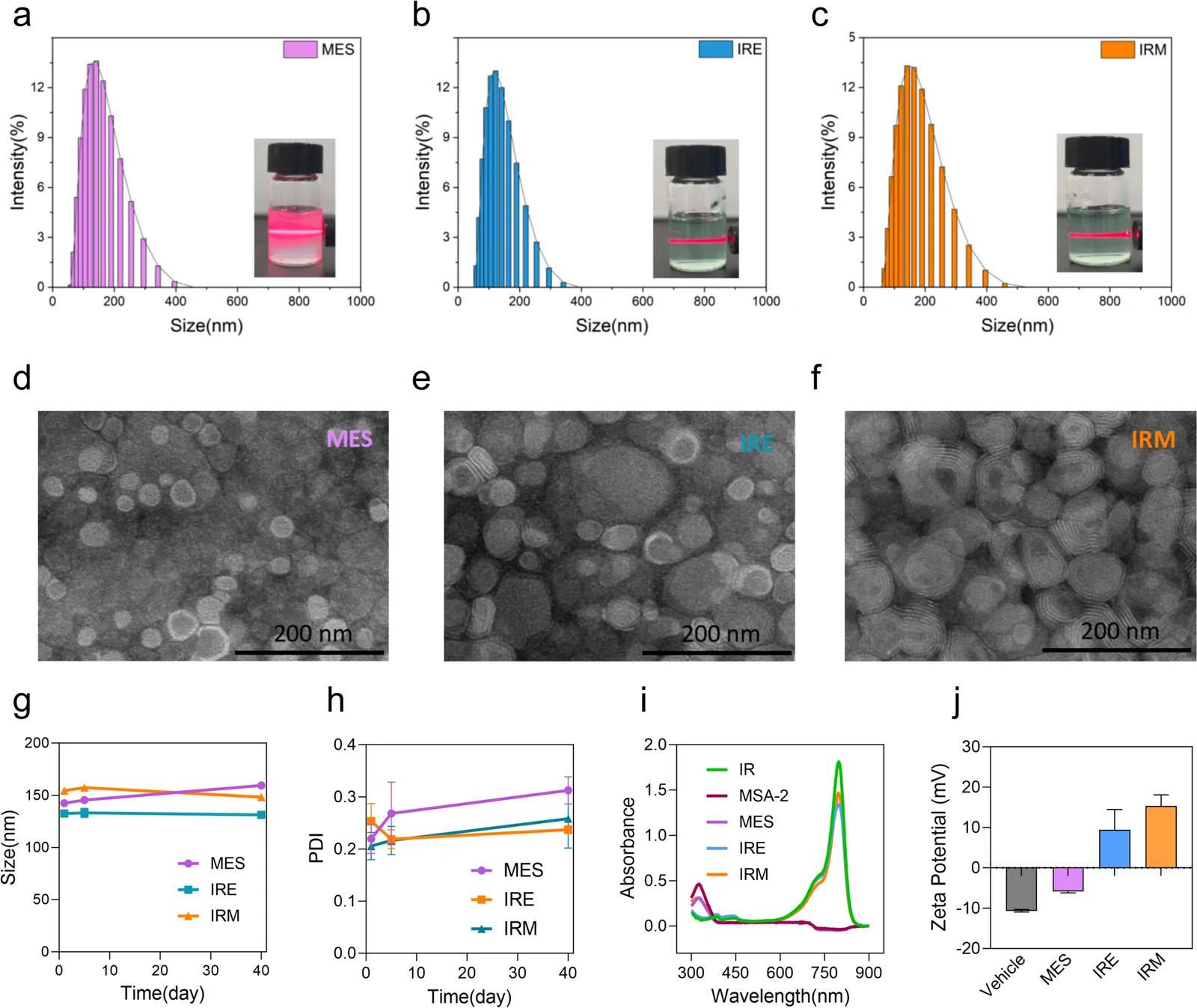
Synthesis and characterization of IRM. (**a**-**c**) Hydrodynamic sizes of MES, IRE and IRM measured by DLS. (**d**-**f**) TEM images of MES, IRE and IRM. (**g**) Size stability of MES, IRE, and IRM over 40 days by DLS. (**h**) PDI stability of MES, IRE, and IRM nanoparticles over 40 days by DLS. (**i**) UV-Vis spectra of solution containing IR, MSA-2, MES, IRE and IRM. (**j**) The zeta potential of Vehicle, MES, IRE, IRM dispersed in water

An initial screening of ethosome carrier (Vehicle) preparation ratios was conducted based on particle size (measured by dynamic light scattering, DLS) and polydispersity index (PDI). Ratios yielding particle sizes within the range of 100–200 nm and a PDI below 0.2 were selected, leading to the identification of Vehicle 2 as the optimal blank carrier for subsequent drug loading (Table.S1). The particle size distribution and morphology of the Vehicle was further characterized (Fig. S2a, c). Subsequently, screening of co-loading ratios of MSA-2 and IR was performed. The particle size, PDI (Table. S2, Fig. S1a-d), and intensity of characteristic ultraviolet absorption peaks—specifically at 327 nm for MSA-2 and 800 nm for IR—were evaluated (Fig. S1e). Furthermore, the morphology of the three IRM formulations with different ratios was examined by TEM. It was observed that both IRM 1 and IRM 3 exhibited irregular shapes and heterogeneous size distributions, whereas IRM 2 displayed a well-defined vesicular structure characteristic of ethosomes with a relatively uniform particle distribution (Fig. S1f-h). Based on these analyses, the IRM 2 ratio was chosen for further preparation. Three types of ethosomal nanoparticles-Nanoplatform loaded solely with MSA-2 (MES), Nanoplatform loaded solely with IR (IRE), and Nanoplatform co-loaded with MSA-2 and IR (IRM) were successfully prepared using the thin-film hydration method. The successful preparation of the nanoparticles was preliminarily verified by the Tyndall effect. The drug loading capacity (DLC%) and encapsulation efficiency (EE%) were also calculated (Table. S3). Although the DLC% of IRM is moderate compared to some lipid-based formulations, this value represents an optimal balance between drug loading and nanoparticle stability. Importantly, the subsequent design of IRM focuses on achieving superior therapeutic efficacy not by maximizing this single parameter, but through a multi-tiered strategy that ensures exceptionally efficient drug delivery and utilization. DLS measurements revealed that MES had a hydrodynamic diameter of 142.43 ± 0.75 nm with a PDI of 0.22 ± 0.03 (Fig. 1a, Table. S4), In comparison, IRE exhibited a size of 132.53 ± 3.31 nm and a PDI of 0.25 ± 0.03 (Fig. 1b, Table. S4), while IRM displayed a size of 154.43 ± 1.21 nm and a PDI of 0.21 ± 0.03 (Fig. 1c, Table. S4). All formulations demonstrated a distinct Tyndall scattering effect upon NIR laser illumination, confirming their colloidal stability. TEM images confirmed that MES, IRE, and IRM exhibited uniform spherical morphology with a characteristic multilamellar vesicle structure (Fig. 1d-f). To evaluate storage stability, all vesicle formulations were dispersed in PBS, (pH 7.4) and stored at 4 °C for 40 days. Stability assessments over this period revealed minimal variations in particle size (< 5% change, Fig. 1g) and PDI (< 10% change, Fig. 1h). UV-vis spectroscopy confirmed successful drug loading in IRM, displaying characteristic absorption peaks at 327 nm (MSA-2) and 800 nm (IR) (Fig. 1i). Zeta potential measurements showed that the vehicle had a surface charge of -10.63 ± 0.09 mV (Figure. 1j), MES had a surface charge of -5.80 ± 0.29 mV, which is lower than that of the Vehicle, confirming the negative charge contributed by MSA‑2. IRE exhibited a positive charge of + 6.55 ± 0.81 mV (Fig. 1j). Notably, IRM resulted in a significantly increased zeta potential (+ 15.37 ± 1.18 mV). This observation suggests that specific molecular interactions occur between IR and MSA-2 within the nanoparticle. Based on an analysis of their chemical structures (Fig. S3a, b), we speculate that potential *π*-*π* stacking between the aromatic domains of IR and MSA-2 may effectively shield the surface exposure of the anionic carboxyl groups of MSA-2, thereby allowing the cationic groups of IR to dominate the particle surface. This surface charge alteration, primarily attributed to the synergistic effects of molecular properties and drug ratios, may contribute to the enhanced cellular interactions of IRM. Previous studies have indicated that positively charged nanoparticles enhance cellular uptake in melanoma [42, 43] and promote internalization, lysosomal escape, and cross-presentation efficiency in BMDCs [44]. Notably, MSA-2 and IR were individually formulated into separate nanoplatforms (MES and IRE, respectively) to eliminate potential confounding effects from the ethosomal carrier and standardize the administration route. Whereas previous studies administered MSA-2 orally [45], its nanoformulation enables injectable delivery, significantly enhancing the scientific rigor of experimental comparisons in subsequent cellular and animal validations.

### Photothermal effect of IRM

**Fig. 2:**
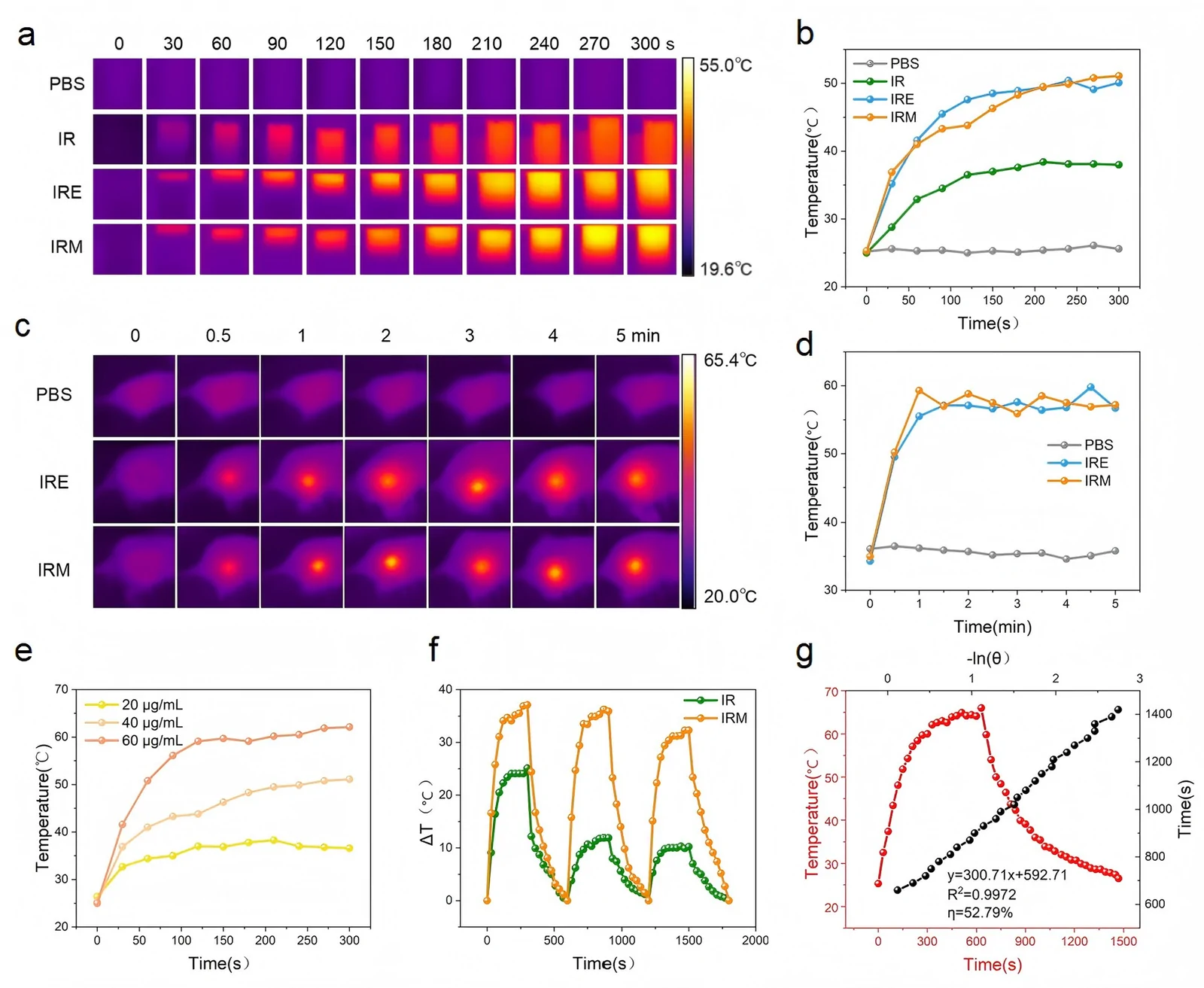
Photothermal effect of IRM. (a) Thermal images of PBS, IR, IRE, and IRM solutions under NIR irradiation (808 nm, 1.0 W/cm^2^). (**b**) Temperature-time curves derived from the thermal images in (**a**). (**c**) Thermal images of PBS, IRE, and IRM solutions under NIR irradiation. (**d**) Temperature-time curves derived from the thermal images in (**c**). (**e**) Concentration-dependent temperature-time curves of IRM solutions. (**f**) recycling-heating curves of IRM solution under NIR irradiation (808 nm, 1.0 W/cm^2^). (**g**) Photothermal conversion efficiency of IRM

In preliminary experiments, an appropriate laser energy level was screened for the PTT of IRM. At 0.5 W/cm², the energy was insufficient to achieve the desired temperature, while at 1.5 W/cm², the temperature rose rapidly and then dropped abruptly, indicating instability and rendering it unsuitable for sustained treatment. (Figure. S4). Consequently, 1.0 W/cm² was selected as the laser energy for subsequent experiments. The photothermal performance of IRM was first evaluated in PBS (pH 7.4) (Fig. 2a, b). Under identical conditions (808 nm, 1.0 W/cm², 5 min), solutions containing 40 µg/mL of IR, IRE, or IRM reached maximum temperatures of 46.2 °C, 50.1 °C, and 51.1 °C, respectively. Both IRE and IRM exceeded the critical PTT threshold of 50 °C. Concentration-dependent studies revealed a gradual temperature increase with rising IRM concentrations, confirming its concentration-responsive photothermal effect (Fig. 2e). Photothermal stability tests demonstrated superior performance of IRM: after three heating cycles, its ΔT decreased by only 4.8 °C, compared with a 14.9 °C decrease for IR (Fig. 2f). Furthermore, the photothermal conversion efficiency (η) of IRM was calculated to reach 52.79% (Fig. 2g). In B16 tumor-bearing mice, IRM-mediated PTT elevated intratumoral temperature from 31.3 °C to 59.1 °C, while laser-only controls showed negligible temperature changes (Fig. 2c, d). These findings confirm that IRM serves as an efficient photothermal agent for PTT-immunotherapy combinations.

### NIR-Ⅱ fluorescence imaging capability of IRM

**Fig. 3:**
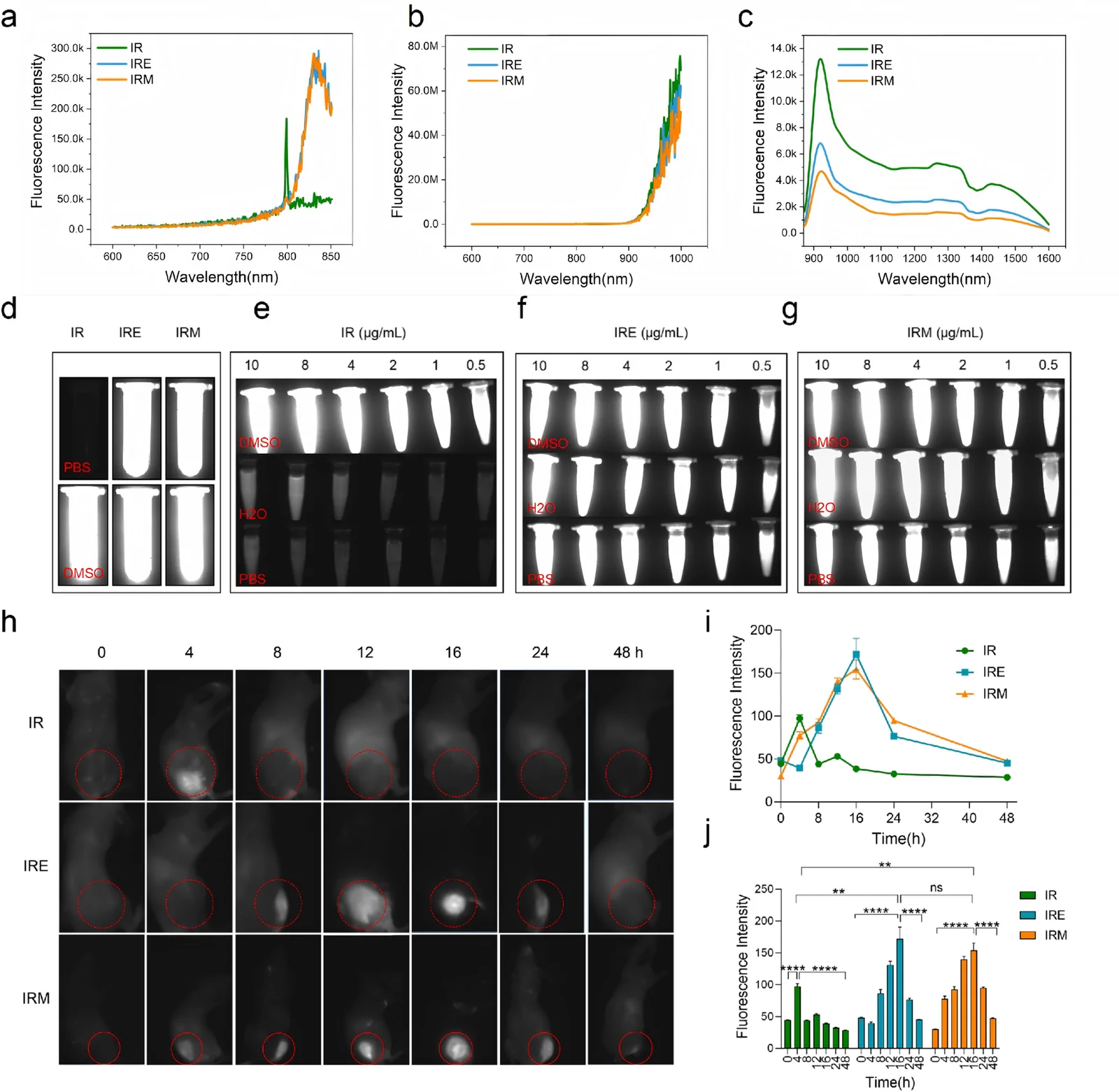
NIR-Ⅱ Fluorescence imaging capability of IRM. (**a**–**b**) Fluorescence spectra of IR, IRE and IRM. (10 µg/mL). (**c**) NIR-Ⅱ spectrum of IR, IRE and IRM (5 µg/mL). (**d**) NIR-Ⅱ fluorescence image of IR, IRE and IRM (10 µg/mL). (e-g) Concentration-dependent NIR-Ⅱ fluorescence imaging of IR, IRE and IRM in DMSO, H_2_O and PBS. (**h**–**j**) Time-dependent NIR-Ⅱ fluorescence imaging of IR, IRE and IRM in vivo. Indicates a group which was compared with other groups. All values are the mean ± SD. *P* values, *p < 0.05, **p < 0.01, ***p < 0.001, ****p < 0.0001

We first systematically evaluated the NIR-Ⅱ fluorescence properties of IR, along with its nano-formulations (IRE and IRM). Fluorescence emission spectral analysis revealed distinct emission peaks in both the NIR-Ⅰ (Fig. 3a, b) and NIR-Ⅱ (Fig. 3c) regions, confirming that IRM possesses dual NIR-Ⅰ and NIR-Ⅱ fluorescence emission characteristics. Comparative NIR-Ⅱ imaging in PBS and DMSO solvents (Fig. 3d) showed that IR exhibited weak fluorescence in PBS but strong signals in DMSO, whereas both IRE and IRM maintained strong fluorescence in both solvents. This discrepancy likely stems from the poor aqueous solubility of IR, which reduces its effective concentration in aqueous media. Nanomaterial formulation (IRE/IRM) improved solubility, leading to a marked enhancement in fluorescence intensity. Concentration-dependent experiments in DMSO, H_2_O, and PBS (Fig. 3e-g) showed a linear increase in fluorescence intensity with concentration for IR, IRE, and IRM, confirming a concentration-dependent response. Notably, the fluorescence intensity of IRM was comparable to that of IRE in all solvents and significantly higher than that of IR. In B16 tumor-bearing mice models (Fig. 3h-j), IR reached the peak of its fluorescence signal at 4 h post-injection and was cleared within 8 h. In contrast, both IRE and IRM peaked at 16 h, clearly delineating tumor margins, with detectable fluorescence signals persisting for up to 24 h. This prolonged retention is attributed to the controlled drug release capability and the enhanced permeability and retention (EPR) effect conferred by the nano-formulations [46]. Taken together, these results establish a solid foundation for the theranostic application of IRM. This study primarily focuses on the functional validation of IRM as a theranostic agent. While a detailed comparison of absolute fluorescence quantum yield presents methodological complexities and is beyond the core scope, the preserved characteristic emission of IR (Fig. 3c) and its demonstrated superior in vivo targeting and imaging performance (Fig. 3h-j) collectively affirm its efficacy as a functional NIR-II imaging module within the IRM nanoplatform. Furthermore, the favorable tumor-targeting capability, prolonged retention at the lesion site, and photo-controlled drug release properties of IRM enable it to serve as an excellent NIR-Ⅱ imaging agent, facilitating real-time, non-invasive tumor visualization and highlighting its significant potential in cancer theranostics. More importantly, this nano-formulation strategy effectively addresses the limitations associated with MSA-2—such as high systemic toxicity and short duration of action [32]—thereby substantially improving the therapeutic safety window and overall efficacy.

### In vitro evaluation of tumor cellular uptake and photothermal toxicity of IRM

**Fig. 4:**
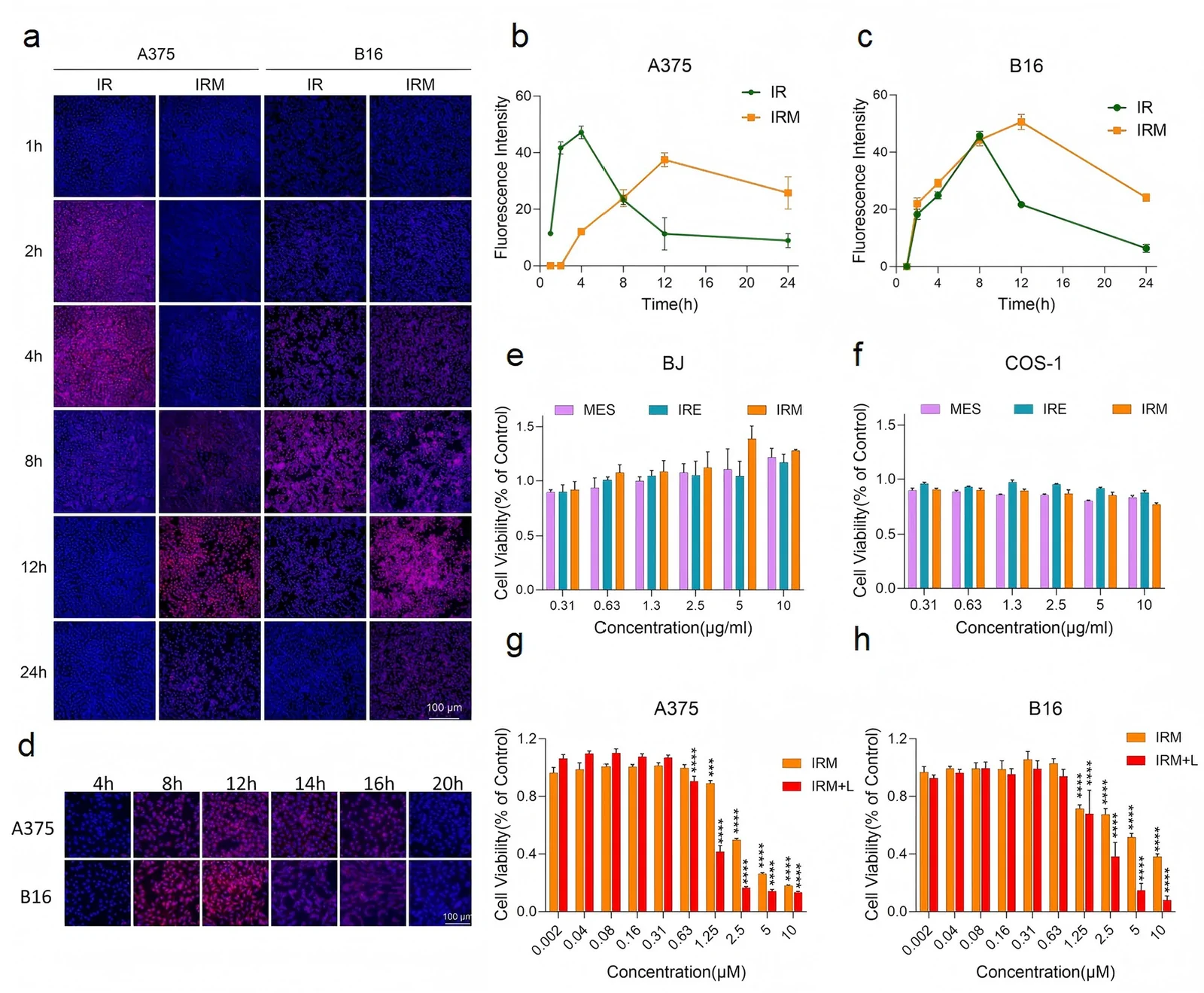
Cellular uptake and cytotoxicity of IRM. (**a**-**c**) Time-dependent cellular uptake of IR and IRM in A375 and B16 cells, Scale bar:100 μm. (**d**) Time-dependent cellular uptake of IRM in A375 and B16 cells, Scale bar:50 μm. (**e-f**) Cell viability of BJ and COS-1 cells after various treatments. (**g-h**) Cell viability of A375 and B16 cells treated with IRM with or without 808 nm laster irradiation. All experiments were carried out with 3 biological replicates. Indicates a group which was compared with other groups. All values are the mean ± SD. *P* values, *P values, *p < 0.05, **p < 0.01, ***p < 0.001, ****p < 0.0001

First, the uptake of nanomedicine by melanoma cells was evaluated. IR emits red fluorescence upon laser excitation, allowing the observation of varying degrees of red fluorescent signals in cells under fluorescence microscopy, which positively correlates with the amount and retention time of the drug internalized by tumor cells. In A375 cells, IR reached peak uptake at 4 h, while IRM peaked at 12 h. In B16 cells, the peak uptake occurred at 8 h for IR and 12 h for IRM, respectively (Fig. 4a-c). This indicates that IRM significantly delays the rate of drug entry into cells. Statistical analysis revealed that at the 12h time point, the fluorescence intensity of IRM was significantly higher than that of IR in both A375 and B16 cells, being 3.31 ± 0.89 times (A375) and 2.34 ± 0.17 times (B16) that of IR, respectively (Fig. S6a, b). Further analysis indicated that after entering the cells, IRM maintained high fluorescence intensity during the 12–16 h period (Fig. 4d, Fig. S5), and a decline in fluorescence was observed only at 18 h. Even at 24 h, detectable fluorescence signals persisted, with the fluorescence intensity of IRM measuring 2.89 ± 0.52 times (A375) and 3.79 ± 0.78 times (B16) that of IR (Fig. S6c, d), further supporting that IRM exhibits stronger efficacy and longer intracellular retention compared to IR. These findings indicate that IRM, as an ethosomal formulation, not only enhances the fluorescence signal intensity of the drug but also significantly delays its entry into tumor cells and prolongs its retention within tumors. More importantly, this alteration in kinetic profile substantially increases the cumulative drug exposure in tumor cells over time, establishing a key “time-dependent compensatory mechanism.” This enables the sustained therapeutic action of the drug delivered by IRM within tumor cells.

Before investigating the photothermal toxicity of IRM on cells, the optimal incubation time was first screened (Fig. S7a, b). After IRM was applied to A375 and B16 cells for 48 h and 72 h, significant cytotoxicity was observed even at very low concentrations, which was not conducive to evaluating the additional effects of laser irradiation. Therefore, 24 h was selected as the optimal duration. Cytotoxicity assays revealed that even at a concentration of 10 µg/mL, IRM did not significantly affect the viability of normal cell lines (BJ and COS-1) (Fig. 4e-f), which is attributed to the lack of targeting toward normal cells. For photothermal efficacy evaluation, A375 and B16 cells were treated with IRM followed by 808 nm laser irradiation (Fig. 4g-h). At a concentration as low as 2.5 µg/mL, IRM alone reduced cell viability to approximately 50%, while the combination of IRM and laser irradiation (IRM + L) resulted in nearly complete cell death, confirming the highly efficient photothermal ablation capability of IRM against melanoma cells. Additionally, the photothermal toxicity of IR and IRE on A375 and B16 cells was also validated (Fig. S7c-f). Consistent with the results observed for IRM, the groups treated with the drugs combined with 808 nm laser irradiation exhibited significantly stronger cytotoxicity compared to groups treated with the same drug concentrations without laser exposure. This confirms the photothermal killing effect of these drugs on tumor cells.

### IRM induces tumor cell pyroptosis

**Fig. 5:**
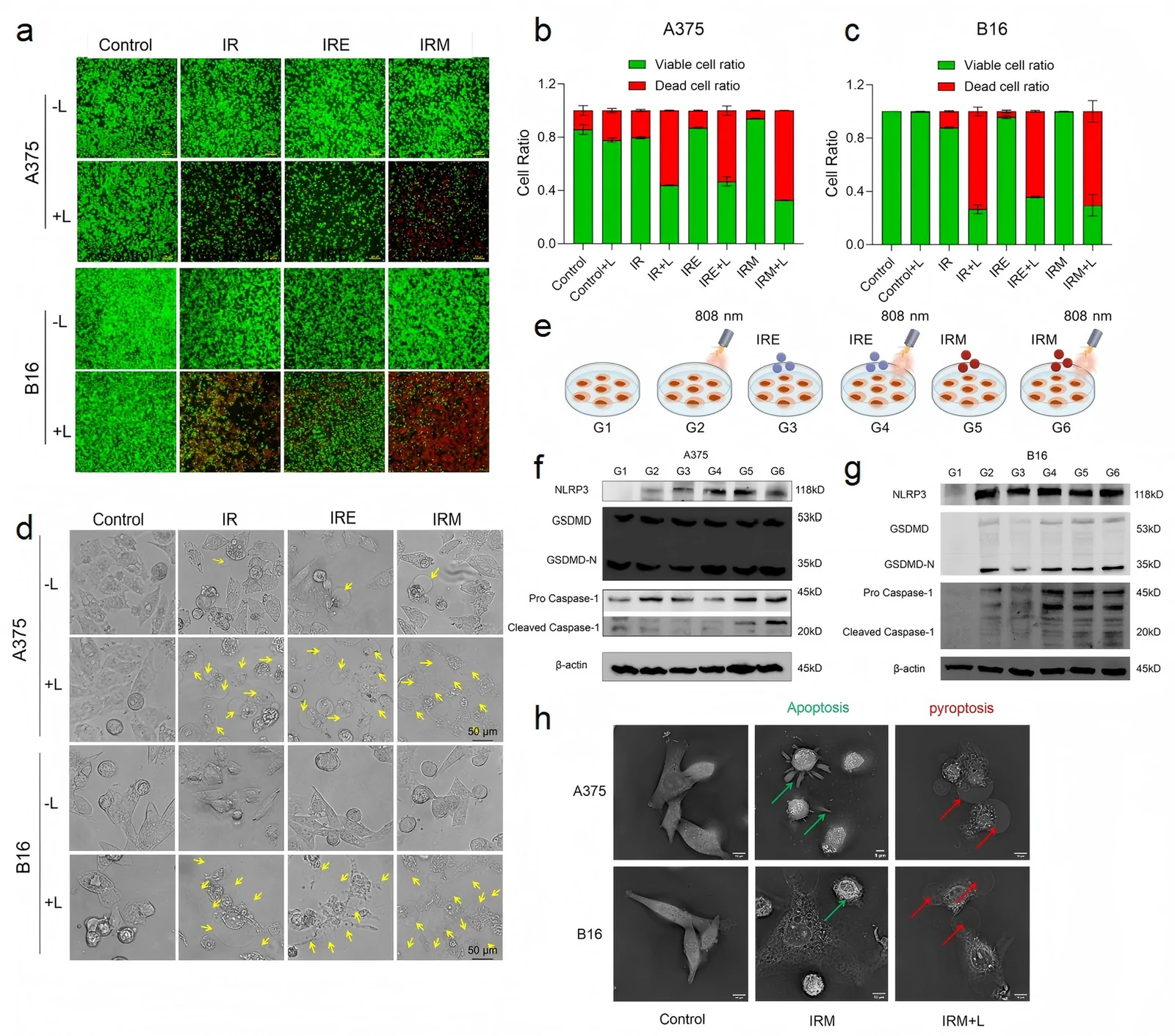
IRM induces pyroptosis in A375 and B16 cells. (**a**) Images of A375 and B16 cells after Calcein-AM/PI staining. Red fluorescence refers to dead cells, and green fluorescence refers to live cells. Scale bar: 100 μm. (**b**, **c**) Quantitative analysis of cell viability corresponding to (**a**). (**d**) Bright-field images of pyroptosis morphology in A375 and B16 cells after IRM with or without laster treatment (808 nm, 1.0 W/cm², 5 min), Scale bar: 50 μm. (**e**) The experimental design of in vitro assays. (**f**, **g**) Western blot of pyroptosis markers in treated A375 and B16 cells. (h) Morphology images of pyroptosis and apoptosis in IRM-treated A375 and B16 cells captured using live cell super-reesolution panoramic microscopy

To further validate the photothermal toxicity of IRM, Calcein AM/PI staining was performed in melanoma cells (Fig. 5a-c, Figure. S8), in which live cells emitted green fluoresce and dead cells showed red fluoresce. The results showed that Neither the Control - L group (blank control) nor the Control + L group (laser‑only control) showed significant cytotoxicity. Compared to the IR plus laser (IR + L) treatment group, both the IRE plus laser (IRE + L) and IRM plus laser (IRM + L) treatment groups demonstrated significant photothermal toxicity against tumors. The IRM + L group showed comparable efficacy to the IR + L group, while the IRE + L group exhibited slightly lower efficacy than the IR + L group, confirming that the nano-formulation did not compromise the photothermal toxicity of IR.

Notably, distinctive “membrane blebbing” was observed in laser-irradiated groups (Fig. 5d), suggesting the possible occurrence of pyroptosis. The core mechanisms of pyroptosis mainly involve three pathways: In the canonical pathway, various danger signals (including PTT, PDT, etc.) activate inflammasomes (such as NLRP3), which then trigger Caspase-1 activation. Caspase-1 cleaves GSDMD to generate its N-terminal domain, forming pores in the cell membrane and releasing pro-inflammatory factors such as IL-1β and IL-18, ultimately leading to pyroptosis. The non-canonical pathway primarily involves activation of caspase-4/5/11, which directly cleaves GSDMD while also inducing K⁺ efflux, thereby activating the NLRP3/caspase-1 pathway. Other pathways involve Caspase-3 activation leading to cleavage of GSDME/GSDMC, or cleavage of GSDMB/GSDME by granzymes A/B (GzmA/B) secreted by immune cells, thereby inducing pyroptosis [11]. Based on these mechanisms and in light of the IRM-plus-laser treatment in this study, we hypothesized that PTT may trigger pyroptosis by activating the NLRP3 inflammasome-C aspase-1-GSDMD pathway to trigger pyroptosis. This process is characterized by pore formation, cellular swelling, and release of proinflammatory cytokine (IL-1β/IL-18), ultimately enhancing antitumor immunity [47]. To investigate this mechanism, Fig. 5e shows the experimental design of in vitro assays. Western blot analysis of pyroptosis-related proteins (GSDMD, Caspase-1, and NLRP3) in A375 and B16 cells revealed the most pronounced expression in the IRM + L group (Fig. 5f, g).

Furthermore, live-cell super-resolution panoramic microscopy was employed to observe pyroptosis phenomena in IRM-treated A375 and B16 cells with higher precision (Fig. 5h, Fig. S9a, b). The experiments demonstrated that, distinct from apoptotic bodies, pyroptotic vesicles exhibit characteristics of large bubbles with thin membranes relative to the overall cell size. These results indicate that IRM not only inhibits tumor growth by promoting apoptosis but also inactivates tumor cells through PTT-induced pyroptosis, collectively achieving synergistic antitumor effects.

### IRM induces antitumor immunity via STING activation

**Fig. 6:**
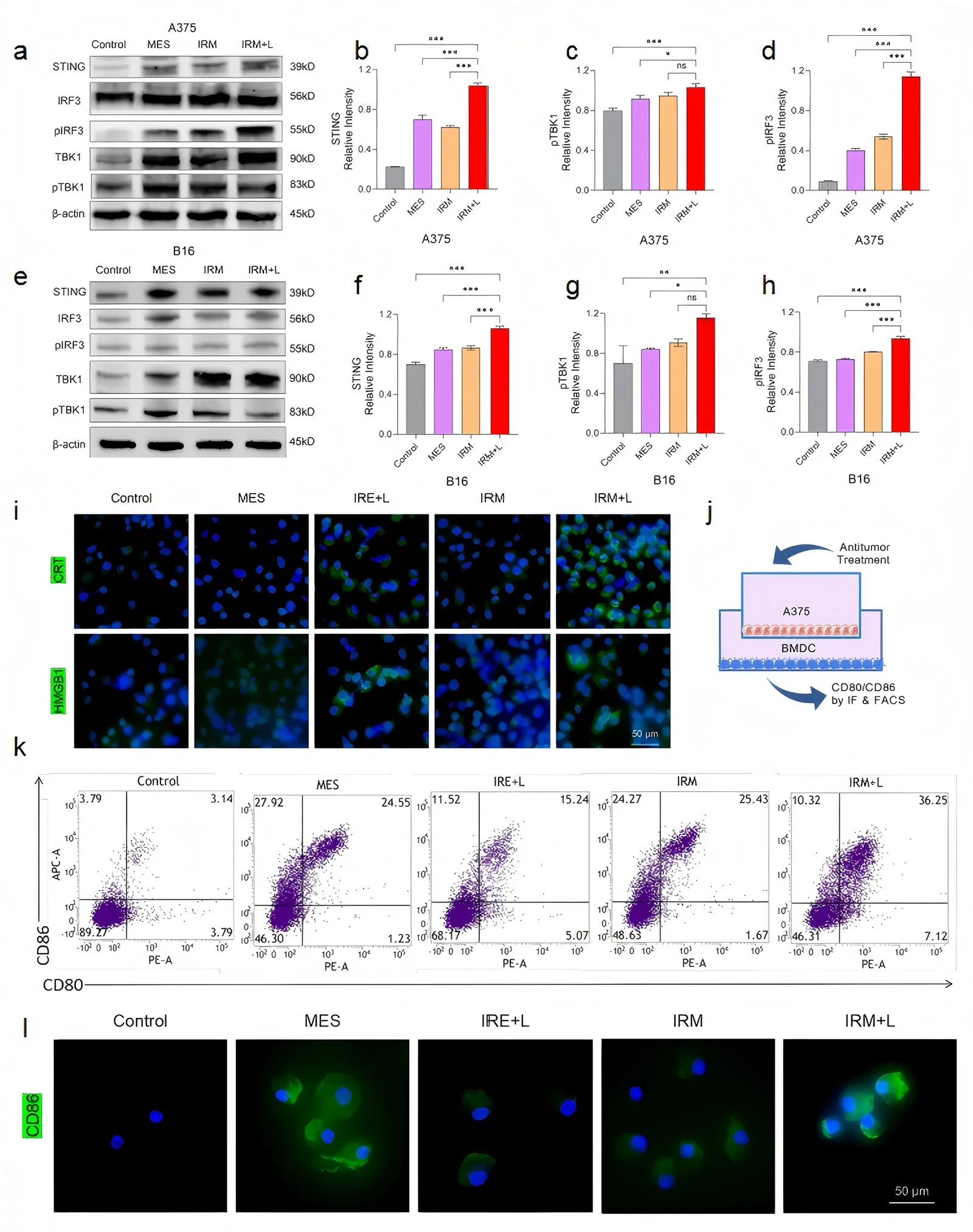
STING activation and immune cell stimulation in vitro. (**a**–**d**) Western blot of STING signaling (STING, pIRF3 and pTBK1) in treated A375 cells. (**e**–**h**) Western blot of STING signaling (STING, pIRF3 and pTBK1) in treated B16 cells. (**i**) The expression of ICD-related molecules (CRT and HMGB1) in A375 cells subjected to different treatments were detected by immunofluorescence. (**j**) Schematic diagram of the co-culture of A375 cells and BMDCs. (**k**) Flow cytometric analysis of BMDC activation (CD80^+^/CD86^+^). (**l**) Immunofluorescence staining of CD86 demonstrates IRM-induced maturation of BMDCs, with nuclei stained blue (Hoechst 33342) and CD86 activation, scale bar: 50 μm. All values are the mean ± SD. *P* values, *p < 0.05, ** *p *< 0.01, ***p < 0.001, ****p < 0.0001

To establish a quantitative link between photothermal effects and STING pathway activation, we examined the release kinetics of the STING agonist MSA-2 from IRM nanoparticles and its downstream signaling. The in vitro release profile showed that MSA-2 release increased over time, plateauing around 12 h post-laser irradiation (Fig. S10a). We first optimized the laser exposure time for IRM-treated A375 and B16 cells. Western blot analysis indicated that 5 min for A375 cells and 10 min for B16 cells were most effective in inducing STING phosphorylation (*p*STING) (Fig. S10b, c, f, g), and these conditions were used for subsequent experiments. Using these optimized durations, we tracked the activation kinetics of the STING pathway. *p*STING levels in both cell lines increased progressively after irradiation, peaking at approximately 12 h (Fig. S10d, e, h, i). This intracellular activation peak (~ 12 h) closely aligned with the plateau phase of MSA-2 release observed in vitro (8–12 h). Together, these synchronized kinetics provide direct evidence that laser-triggered release of MSA-2 from IRM drives STING pathway activation. Building upon this established causal chain, we next sought to validate the activation of the STING pathway and immune cells in vitro, we first examined the expression levels of key proteins in the canonical STING signaling pathway using western blot analysis. The results showed that in IRM-treated A375 and B16 cells, the expression of STING, *p*TBK1, and pIRF3 was significantly upregulated compared to the control group (Fig. 6a-h). Notably, the phosphorylation levels of *p*TBK1 and *p*IRF3 were markedly enhanced, confirming that IRM effectively activated the STING pathway. Additionally, immunofluorescence was performed to assess ICD in IRM-treated tumor cells by examining the DAMPs markers HMGB1 and CRT (Fig. 6i). The results indicated that the IRM + L treatment group induced nucleo-cytoplasmic translocation of HMGB1 (evidenced by green fluorescence shifting from the nucleus to the cytoplasm and membrane) and membrane translocation of CRT (punctate green fluorescence on the cell membrane), confirming that IRM activated ICD in vitro. Furthermore, A375 cells subjected to different treatments were co-cultured with BMDCs (Fig. 6j). The activation status of BMDCs was evaluated by flow cytometry (Fig. 6k) using the expression of co-stimulatory molecules (CD80^+^/CD86^+^) as activation markers. The results demonstrated that the MES and IRM groups induced a comparable degree of immune cell activation, while the effect of IRE + L was relatively weaker. This indicates that both STING activation alone and photothermally-induced ICD can stimulate dendritic cell maturation, but STING activation exhibits a stronger effect than ICD. The similar effects observed in the IRM and MES groups suggest that, in the absence of laser irradiation, the primary immunostimulatory component in IRM is MSA-2. In contrast, the IRM + L group showed a significantly higher proportion of CD80^+^/CD86^+^ double-positive BMDCs compared to the IRE + L and IRM groups, indicating that the combination of PTT and STING activation synergistically enhanced immune cell activation. Immunofluorescence analysis of the co-cultured BMDCs (Fig. 6l) supported the flow cytometry results, with the IRM + L group exhibiting the strongest green fluorescence and the most potent immune activation. In summary, IRM effectively activates immune cells in vitro, and the combination of PTT and STING signaling results in significantly enhanced immunostimulatory effects.

### In vivo antitumor effect and immune activation of IRM

**Fig. 7:**
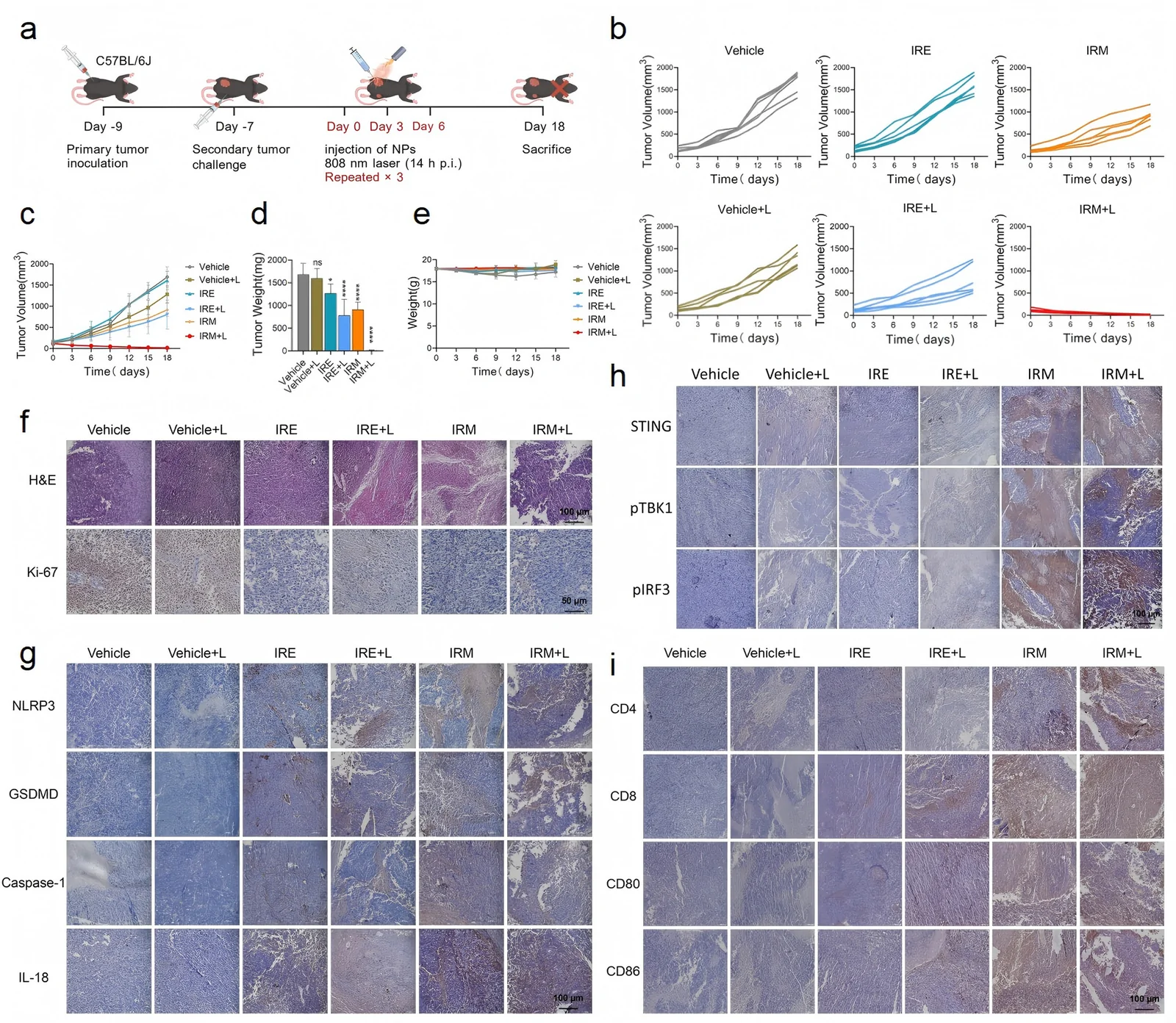
Primary tumor therapeutic efficacy and immune activation in vivo. (**a**) Schematic illustration of the in vivoin vivo experimental schedule. (**b**) Individual growth curves of primary tumor for each treatment group (Vehicle, Vehicle+L, IRE, IRE+L, IRM, IRM+L. (**c**) Growth curves of primary tumor for different group. (**d**) Terminal tumor weights. (**e**) Body weight curves of mice. (**f**) H&E and Ki-67 staining of primary tumors. (**g**) IHC analysis of pyroptosis-related proteins in primary tumors, scale bar: 100 μm. (**h**) IHC staining of STING activation in primary tumors, scale bar: 100 μm. (**i**) IHC detection of immune activation in primary tumors, scale bar: 100 μm. Indicates a group which was compared with other groups. All values are the mean ± SD. *P* values, *p < 0.05, **p < 0.01, ***p < 0.001, ****p < 0.0001

In female C57BL/6J mice, B16 cells were subcutaneously injected bilaterally to establish an primary tumor model and a distant metastasis model (primary and distant foci, simulating tumor metastasis) to evaluate the in vivo anti-tumor efficacy and systemic immune activation of IRM (Fig. 7a). Treatment of the primary tumors began when their volume reached 100–200 mm³. Different drug formulations were intratumorally injected into the primary tumors, followed by 808 nm laser irradiation (1.0 W/cm², 5 min) after 14 h, for three treatment cycles. Over an 18-day experimental period, mouse body weights and tumor volumes were recorded every two days. At the end of the treatment cycle (corresponding to day 7), blood was collected for biochemical and hepatorenal function tests to assess the in vivo biosafety of the nanoparticle. On day 18, the mice were euthanized, and major organs and tumor tissues were collected for analysis. According to the recorded results, the primary tumors in the control and laser-only groups showed significant growth, with volumes increasing to 11.46 and 9.79 times their initial sizes by day 18, respectively. Compared with other groups, the IRE group exhibited only a 24.19% tumor inhibition rate (relative to the control group), while the IRE + L group showed a 52.39% anti-tumor effect, twice that of the IRE group, attributable to the anti-tumor effect of PTT. The IRM group achieved a tumor inhibition rate of only 45.88%, whereas the IRM + L group achieved a rate exceeding 95% (Fig. 7b-d), demonstrating the multi synergistic anti-tumor effects of PTT combined with STING immune-mediated responses. Body weight changes of all mice were monitored throughout the treatment period (Fig. 7e). No significant weight loss observed, indicating the high biosafety of the treatment. H&E staining of major organs in mice also revealed no obvious abnormalities (Fig. S12a), and hemolysis assay results (Fig. S12b, c) showed that the hemolysis rates in all treatment groups were comparable to those in the PBS group (negative control), indicating that the nanoparticle did not cause hemolysis in mice. Additionally, hematological parameters in the drug-administered groups showed no significant differences from the blank control group, with no abnormalities observed (Fig. S11d-f). demonstrating the favorable in vivo biosafety profile of the nanoparticle. Hematoxylin and eosin (H&E) staining and Ki-67 staining of the harvested primary tumors showed extensive tumor cell destruction in the IRM + L group, along with the weakest Ki-67 signal, indicating significant anti-tumor efficacy (Fig. 7f). To evaluate longer-term biosafety, we extended the observation period from 18 days to 45 days. The results showed that the IRM + L group achieved a 45-day survival rate of 76.19%, which was significantly better than that of the Vehicle group (Fig. S12a). Terminal histopathological analysis (Fig. S12b) and blood biochemical assays (Fig. S12c-h) indicated no damage to major organs in the IRM + L group, with liver and kidney function markers remaining within normal ranges, demonstrating favorable intermediate-term safety of this treatment regimen.

To systematically elucidate the immunomodulatory mechanisms responsible for the observed antitumor effects, we performed a comprehensive analysis of immune-related markers in both primary and distant tumors. Immunohistochemical analysis of immunogenic cell death (ICD) markers (HMGB1 and CRT) in tumor tissues revealed that both the IRE + L and IRM + L groups exhibited significantly stronger signals than other groups (Fig. S13), suggesting that PTT induced ICD in vivo. Additionally, the expression of PTT - induced pyroptosis-related proteins in primary tumors was detected (Fig. 7g). The results showed that the IRE + L and IRM + L groups displayed stronger fluorescence intensity compared to non-laser groups, confirming that PTT triggered pyroptosis in tumor tissues. Similarly, detection of STING pathway-related proteins in primary tumor tissues (Fig. 7h) demonstrated significantly higher expression in the IRM and IRM + L groups than in other groups. The IRM + L group showed stronger signal intensity than the IRM group, proving the release of DAMPs and the induction of pyroptosis triggered by photothermal therapy (PTT), synergizing with the STING agonist, collectively enhance STING-mediated immune activation in tumor tissues compared to relying on MSA-2 alone for its immunostimulatory effect. The activation levels of antigen-presenting cells (APCs, mainly CD80^+^and CD86^+^) and T cells (mainly CD4^+^and CD8^+^) in primary tumors were also examined (Fig. 7i). The IRM + L group exhibited the strongest fluorescence intensity, indicating enhanced immune activation in tumors through the synergy between PTT and STING-mediated immunity. These results demonstrate that the combination of IRM and PTT can induce both ICD and pyroptosis, significantly inhibit primary tumor growth, activate immune responses through PTT, further enhance STING-mediated immunity, and promote the activation of APCs and infiltration of T cells within tumor tissues.

**Fig. 8:**
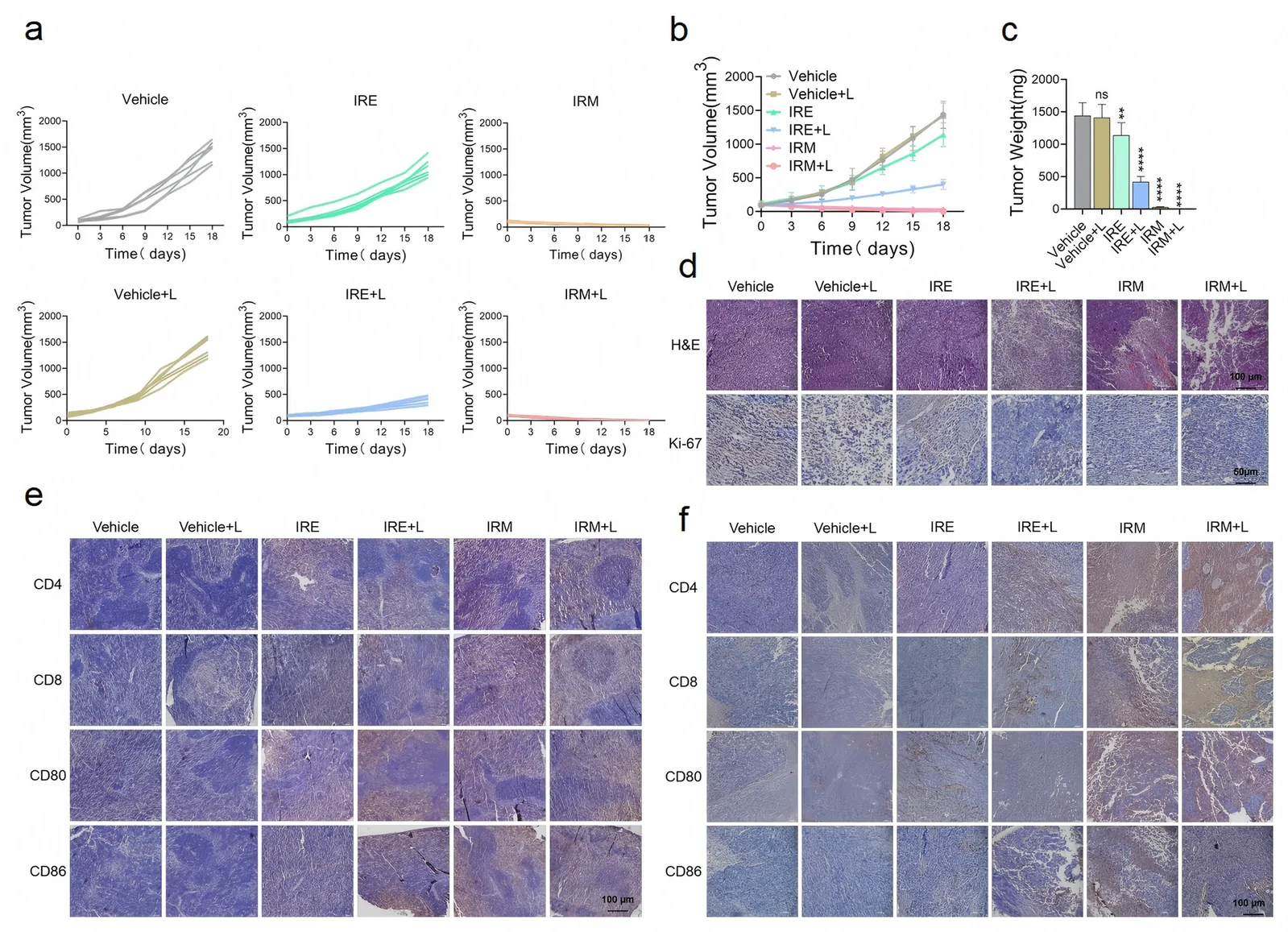
Distant tumor regression and systemic immune cell activation. (**a**) Individual growth curves of distant tumor for each treatment group. (**b**) Growth curves of the distant tumor. (**c**) Tumor terminal weights. (**d**) H&E and Ki-67 staining of distant tumors, scale bar: 100 μm. (**e**) IHC analysis of immune cell activation in spleen, scale bar: 100 μm. (**f**) IHC analysis of DCs and T cell activation in distant tumors. indicates a group which was compared with other groups. All values are the mean ± SD. *P* values, *p < 0.05, ** *p *< 0.01, ***p < 0.001, **** *p* < 0.0001

Similarly, the anti-tumor efficacy in distant tumors was evaluated. As shown by the tumor growth curves and volume statistics (Fig. 8a, b), the distant tumors in the vehicle and laser-only groups exhibited significant growth, with volumes increasing to 16.07 and 13.81 times their initial sizes by day 18, respectively. The IRE + L group exhibited a significantly higher distant tumor inhibition rate than the IRE group. This difference is hypothesized to result from the combined immune activation triggered by PTT-induced ICD and pyroptosis. Correspondingly, the IRM + L group demonstrated the highest distant tumor inhibition rate, which is attributed to the synergistic enhancement of STING immune activation by PTT-induced pyroptosis and ICD, collectively stimulating anti-tumor immunity in distal tumors and effectively suppressing their growth. The tumor weights measured after dissection were consistent with the trends in tumor volume changes (Fig. 8c), both indicating effective suppression of distant tumors in the IRM + L group. To validate systemic immune activation, spleens from mice in each treatment group were collected to assess T cell activation markers (CD4, CD8). The results revealed significantly elevated expression in the IRE + L, IRM, and IRM + L groups (Fig. 8e), confirming that both PTT and STING activation contribute to systemic immune activation. Additionally, the expression levels of T cells and APCs in distant tumor tissues were examined (Fig. 8f). The results indicated markedly higher expression in the IRM + L group, consistent with the observed significant inhibition of distant tumors. H&E staining and Ki-67 staining of distant tumors further demonstrated the superior anti-tumor efficacy of the IRM + L group (Fig. 8d).

To further elucidate the immunomodulatory effects of IRM on the TME, particularly on suppressive cell populations, we performed multiplex immunofluorescence analysis on tumor tissue sections (Fig. S14). The results demonstrate that IRM treatment significantly reshaped the immune landscape of the tumors. Notably, we observed a marked increase in infiltrating CD8⁺ T cells alongside a relative decrease in Foxp3⁺ regulatory T cells (Tregs) in both primary and distant tumors, suggesting a shift toward a more immunopermissive and active TME. Furthermore, a pronounced reduction in CD206⁺ M2-type tumor-associated macrophages was evident, further confirming the reversal of the immunosuppressive state. This suggests that the combination of PTT and STING immune activation orchestrates a potent systemic anti-tumor responses, which remodels the immune microenvironment of distant tumors by not only enhancing effector T-cell infiltration but also concomitantly reducing immunosuppressive cell populations This dual modulation effectively reverses immunosuppression and inhibits distant tumor growth.

### Validation of STING-dependent antitumor efficacy and immune activation of IRM in *STING*-KO mice

**Fig. 9:**
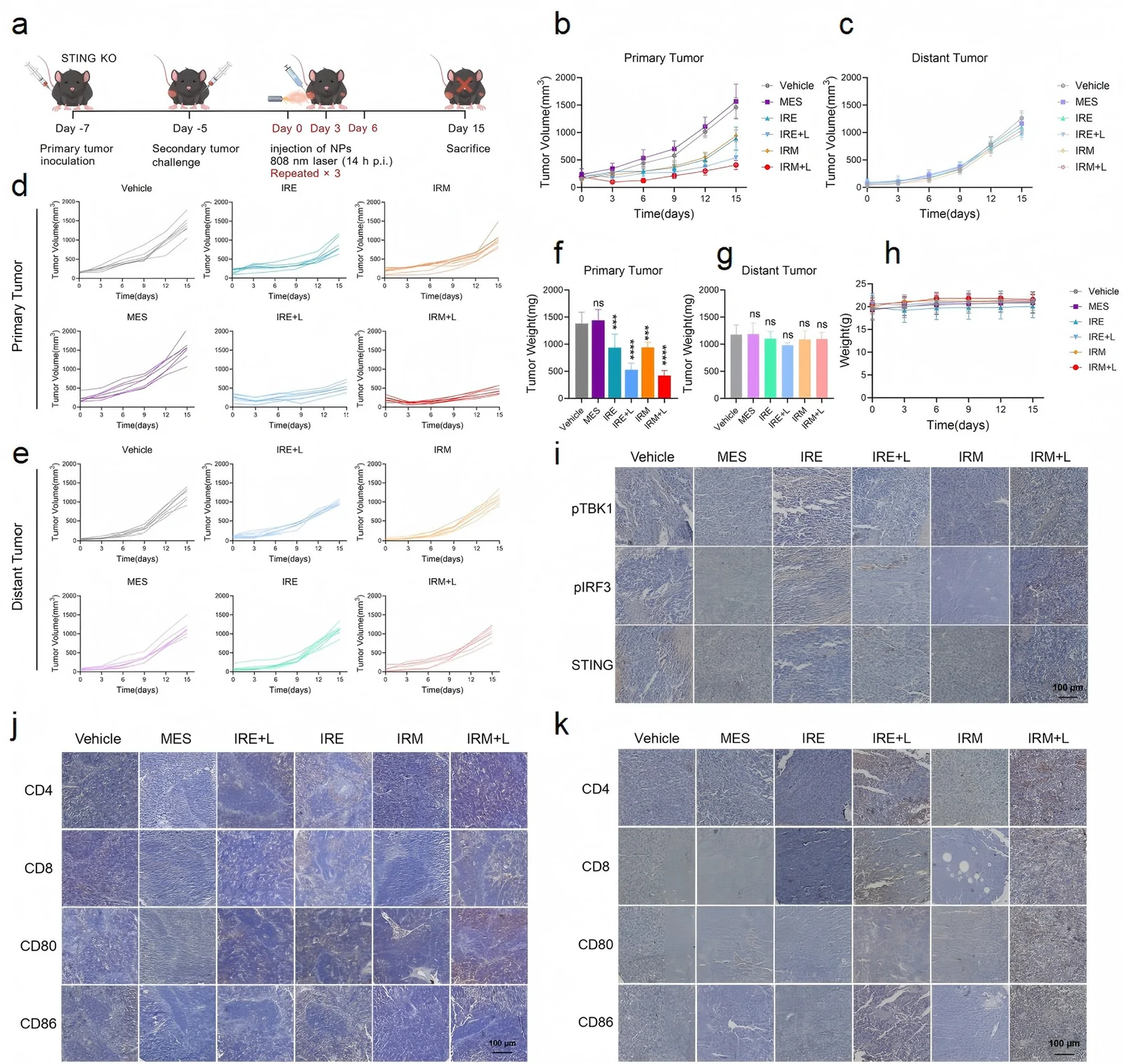
Tumor therapeutic efficacy and immune activation in *STING*-KO mice. (**a**) Schematic illustration of the in vivo experimental schedule. (**b**) Growth curves of primary tumor in mice after different treatment. (**c**) Gorwth curves of distant tumors in mice after different treatment. (**d**) Individual growth curves of primary tumors for each treatment group (Vehicle, MES, IRE,IRE+L,IRM, IRM+L). (**e**) Individual growth curves of distant tumors for each teratment group (Vehicle, MES, IRE, IRE+L, IRM, IRM+L). (**f**) Primary tumor terminal weights. (**g**) Distant tumor terminal weights. (**h**) Body weight changes in mice. (**i**) IHC staining of STING activation in primary tumors, scale bar: 100 μm. (**j**) IHC analysis of immune cell activation in spleen scale bar: 100 μm. (**k**) IHC detection of immune activation in distant tumors, scale bar: 100 μm. Indicates a group which was compared with other groups. All values are the mean ± SD. *P* values, *p < 0.05, **p < 0.01, ***p < 0.001, ****p < 0.0001

To validate the role of STING-mediated immunity in IRM, a subcutaneous dual-tumor model (primary/distant) was established in *STING*-KO mice to evaluate the tumor inhibitory effects and immune activation of each treatment group under STING pathway inactivation (Fig. 9a). After modeling, the same treatment protocol was applied, and mouse body weight and tumor volume were recorded every 2 days for 15 days. The results showed that primary tumors in both the vehicle and MES groups grew significantly, with volumes increasing to 9.01 and 6.87 times their initial sizes by day 15, respectively. The IRE + L and IRM + L groups exhibited primary tumor inhibition rates of 61% and 69%, respectively, with comparable efficacy (Fig. 9b, d). This phenomenon is attributed to the inability of MSA-2 in IRM to activate the STING pathway after gene KO, indicating that the antitumor effects in these two groups solely originated from PTT-induced ICD and pyroptosis. In contrast to primary tumors, distant tumors in all groups showed similar volumes and significant growth, with no notable inhibition compared to the vehicle (Fig. 9c, e). The weights of dissected tumors were consistent with the tumor volume trends (Fig. 9f, g), and no significant fluctuations in body weight were observed within 15 days, confirming the absence of substantial toxicity in *STING*-KO mice (Fig. 9h). Immunohistochemical analysis of primary tumors revealed no fluorescent signals of STING pathway-related proteins in any group (Fig. 9i), indicating the absence of STING immune activation. ICD detection in primary tumors showed fluorescent signals in the IRE + L and IRM + L groups, while other groups were similar to the vehicle (Fig. S15). No obvious fluorescence was detected for CD4^+^/CD8^+^ T cells or CD80^+^/CD86^+^ APCs in the spleen and distant tumors (Fig. 9j, k), consistent with the lack of inhibition in distant tumors. Analysis revealed that ICD induced by PTT alone without STING immune synergy failed to effectively activate systemic immunity to suppress distant tumor growth. This inversely demonstrates the critical role of PTT and STING immune synergy in enhancing systemic immune activation and inhibiting distant tumors.

## Conclusion

In this study, we successfully developed an immunometabolic nanoplatform, IRM, Which integrates real-time NIR-Ⅱ imaging, highly efficient photothermal therapy, and STING-driven immunomodulation into a unified system for melanoma theranostics. IRM not only ablates tumors by inducing pyroptosis and ICD through its outstanding photothermal performance (η = 52.79%), but also remodels the tumor microenvironment and elicits a systemic immune response via the synergistic “PTT–STING” mechanism. Although IRM exhibits a moderate drug-loading capacity, its remarkable therapeutic efficacy stems from a multi-level synergistic strategy: at the delivery level, it achieves tumor-targeted retention, delayed cellular uptake, and NIR-triggered on-demand drug release; at the mechanistic level, photothermal stress-induced ICD and MSA‑2-mediated STING activation form a positive feedback loop, mimicking the immune-priming and amplification logic of advanced sequential drug delivery systems [33, 34]. Studies in *STING*‑KO models definitively confirm that this synergy is crucial for eliciting the abscopal effect against distant tumors. The multimodal synergistic concept proposed in this work aligns with current cutting-edge directions that employ composite targeting strategies—such as active recognition coupled with chemical anchoring [48], or active targeting combined with enzymatic aggregation [49] to enhance therapeutic precision. Future work will further optimize the imaging performance and explore the clinical translation potential of this platform. In summary, by integrating metabolic targeting, deep-tissue imaging, and immune synergy, IRM provides a new paradigm for overcoming metastatic melanoma and lays the foundation for the development of next-generation precision.

## Supplementary Information

Below is the link to the electronic supplementary material.

Supplementary Materials

## Data Availability

Data will be made available on request.

## Abbreviations

IRM: Ethosomes co-loading photosensitizer IR-817 and STING agonist MSA-2
IRE: Ethosomes singly loading photosensitizer IR-817
MES: Ethosomes singly loading STING agonist MSA-2
IR: Free single-agent IR-817
PTT: Photothermal Therapy
OATPs: Organic Anion Transporting Polypeptides
ICD: Immunogenic Cell Death
DAMPs: Damage-Associated Molecular Patterns
DLS: Dynamic Light Scattering
PDI: Polydispersity Index
TEM: Transmission Electron Microscopy
NIR: Near-Infrared
FBS: Fetal Bovine Serum
PBS: Phosphate-Buffered Saline
MTT: 3-(4,5-diMethylthialzol-2-yl)-2,5-diphe-nyltetrazolium bromide
TME: Tumor Microenvironment
SPC: Soybean Phospholipids
IHC: Immunohistochemistry
IF: Immunofluorescence
BMDCs: Bone Marrow-Derived Dendritic Cells
APCs: Antigen-Presenting Cells
DCs: Dendritic Cells
