# Correction: A NIR-Ⅱ-Immunostimulatory nanoplatform rewires immunometabolism to unleash STING-driven antitumor immunity

**DOI:** 10.1186/s12951-026-04904-2

**Published:** 2026-08-22

**Authors:** Xun Yang, Xuefeng Chen, Minhao Chen, Simei Yang, Ya Wu, Hongye Liao, Tong Xia, Gaoyang Shen, Changzhen Sun, Li Liu

**Affiliations:** 1 Skin Structure and Function Key Laboratory of Luzhou, Department of Dermatology, The Affiliated Hospital, Southwest Medical University, Sichuan Province 646000, Luzhou, China; 2 Drug Research Center of Integrated Traditional Chinese and Western Medicine, The Affiliated Traditional Chinese Medicine Hospital, Southwest Medical University, Sichuan Province 646000, Luzhou, China; 3 Department of Vascular Surgery, The Affiliated Hospital,, Southwest Medical University, Sichuan Province 646000, Luzhou, China; 4 Luzhou Key Laboratory of Research and Development of Medical Institution Preparations and Large-scale Health Products, The Affiliated Traditional Chinese Medicine Hospital, Southwest Medical University, Luzhou, Sichuan Province 646000 China

**Correction: Journal of Nanobiotechnology (2026) 24:348**

10.1186/s12951-026-04162-2

In this article, the authors would like to replace two inadvertently duplicated images in Fig. 3H with the correct 8-hour images and do not affect the study’s data analysis, results, or conclusions. The old incorrect and correct versions are displayed below.

Incorrect Fig. 3.

**Figure:**
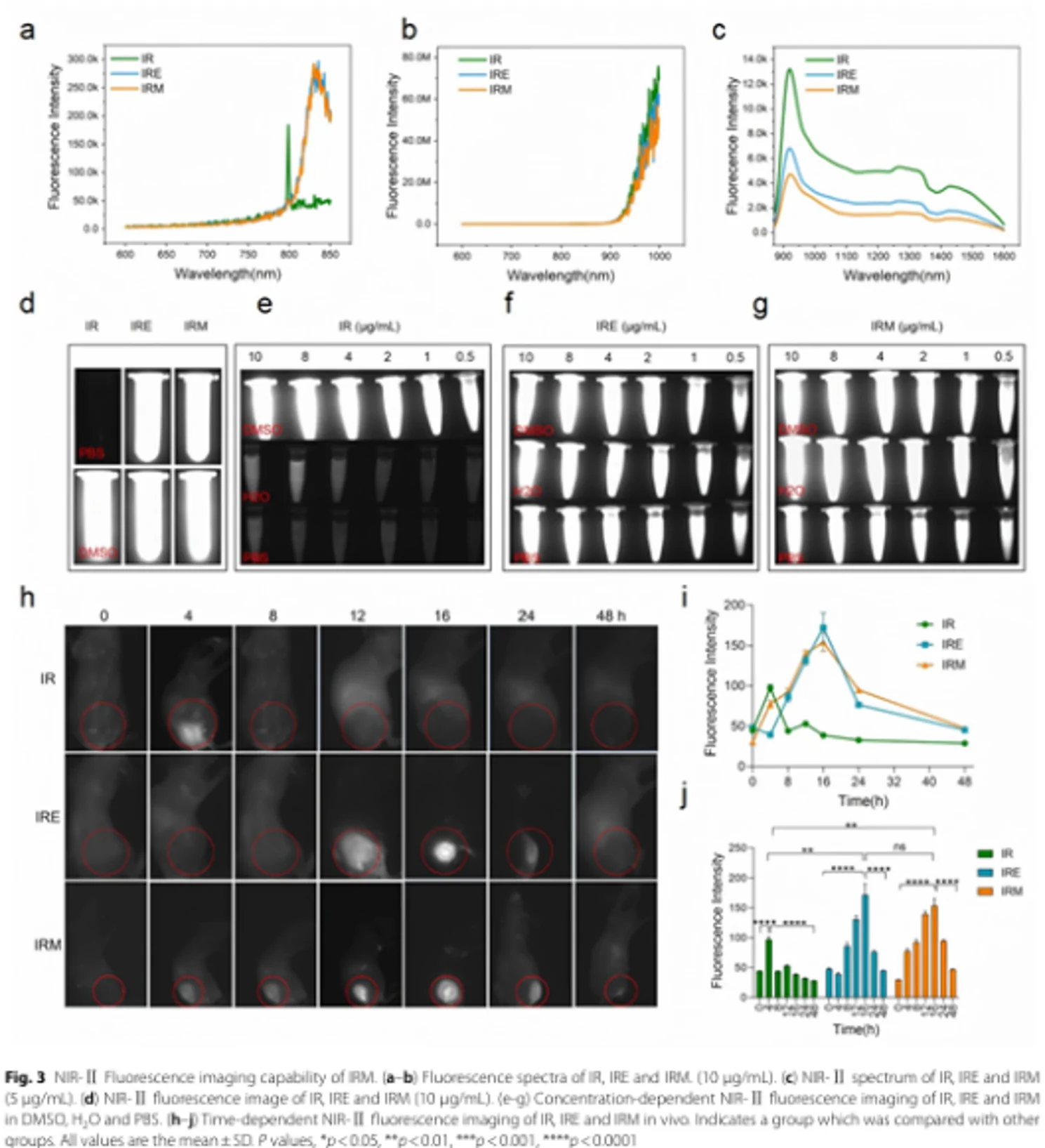

Correct Fig. 3.

**Fig. 3:**
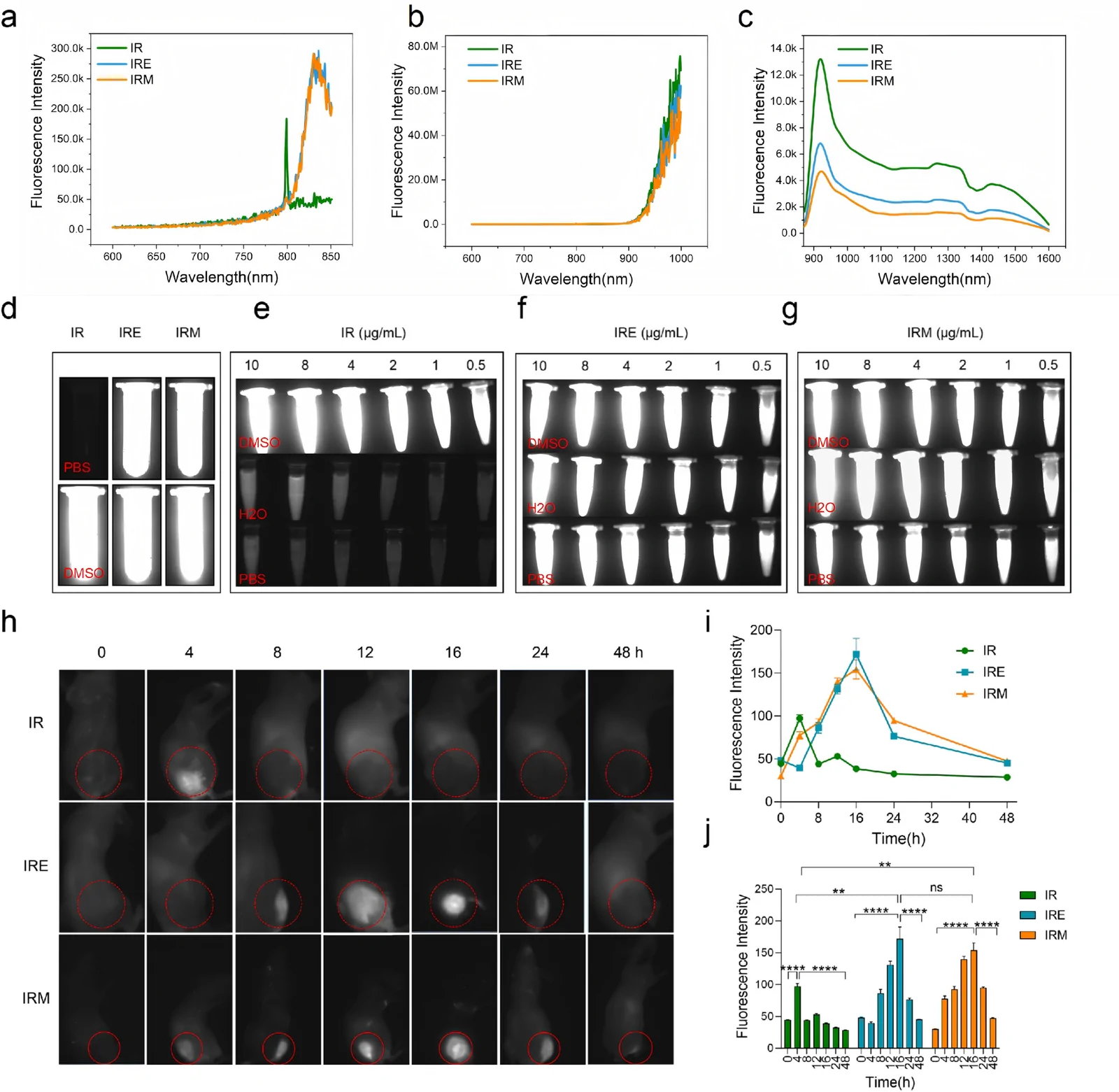
NIR-Ⅱ Fluorescence imaging capability of IRM. (**a**–**b**) Fluorescence spectra of IR, IRE and IRM. (10 μg/mL). (**c**) NIR-Ⅱ spectrum of IR, IRE and IRM (5 μg/mL). (**d**) NIR-Ⅱ fluorescence image of IR, IRE and IRM (10 μg/mL). (e-g) Concentration-dependent NIR-Ⅱ fluorescence imaging of IR, IRE and IRM in DMSO, H2O and PBS. (**h**–**j**) Time-dependent NIR-Ⅱ fluorescence imaging of IR, IRE and IRM in vivo. Indicates a group which was compared with other groups. All values are the mean±SD. *P* values, *p<0.05, **p<0.01, ***p<0.001, ****p<0.0001

The original article has been corrected.

