# Supplementary material for: A NIR-Ⅱ-Immunostimulatory nanoplatform rewires immunometabolism to unleash STING-driven antitumor immunity
Source: J Nanobiotechnology. 2026 Mar 15;24:328. doi: 10.1186/s12951-026-04162-2 (PMC13059612; doi:10.1186/s12951-026-04162-2)
Supplement: Supplementary file 1 — Supplementary Materials [file 12951_2026_4162_MOESM1_ESM.docx]

**A NIR-II-Immunostimulatory Nanoplatform Rewires Immunometabolism to Unleash STING-Driven Antitumor Immunity**

Xun Yang^a,1^, Xuefeng Chen^a,1^, Minhao Chen^a^, Simei Yang^b^, Ya Wu^c^, Hongye Liao^a^, Tong Xia^a^, Gaoyang Shen^a^, Changzhen Sun^b,d#^, Li Liu^a#^.

^a^ Skin Structure and Function Key Laboratory of Luzhou, Department of Dermatology, The Affiliated Hospital, Southwest Medical University, Sichuan Province, 646000，Luzhou, China.

^b^ Drug Research Center of Integrated Traditional Chinese and Western Medicine, The Affiliated Traditional Chinese Medicine Hospital, Southwest Medical University, Sichuan Province, 646000，Luzhou, China.

^c^ Department of Vascular Surgery, The Affiliated Hospital of Southwest Medical University, Sichuan Province, 646000，Luzhou, China.

^d^ Luzhou Key Laboratory of Research and Development of Medical Institution Preparations and Large-scale Health Products, The Affiliated Traditional Chinese Medicine Hospital, Southwest Medical University, Sichuan Province, 646000，Luzhou, China.

**^#^Correspondence author:**

Li Liu

Email: liuli@swmu.edu.cn

**^#^Co-Correspondence author:**

Changzhen Sun

Email:[sunchangzhen@swmu.edu.cn](mailto:sunchangzhen@swmu.edu.cn)

**Keywords:**
NIR-II imaging; Photothermal therapy; STING pathway; Pyroptosis; Immunometabolic reprogramming

| **Table. S1.** Particle size and PDI of blank vehicles at different composition ratios. | | | |
| --- | --- | --- | --- |
| Formulation | Composition Ratio | Size (nm, DLS) | PDI (DLS) |
| Vehicle 1 | 5:1 (W:V) | 177.7 ± 2.5 | 0.232 ± 0.017 |
| Vehicle 2 | 10:1 (W:V) | 163.7 ± 0.9 | 0.236 ± 0.029 |
| Vehicle 3 | 15:1 (W:V) | 184.5 ± 3.8 | 0.252 ± 0.028 |
| Vehicle: Blank carrier (soybean phospholipids + 20% EtOH aqueous solution).  Ratio notation: For Vehicles, "W:V" represents weight (mg) to volume (mL) ratio.  Data represent mean ± standard deviation (n = 3). | | | |

| **Table. S2.** Exploration of different ratios of MSA-2 and IR in the nanoplatform. | | | |
| --- | --- | --- | --- |
| Formulation | Composition Ratio | Size (nm, DLS) | PDI (DLS) |
| IRM 1 | 1:1 (MSA-2:IR, W:W) | 161.0 ± 3.9 | 0.205 ± 0.083 |
| IRM 2 | 1:2 (MSA-2:IR, W:W) | 143.6 ± 3.6 | 0.177 ± 0.036 |
| IRM 3 | 1:3 (MSA-2:IR, W:W) | 148.5 ± 2.0 | 0.230 ± 0.041 |
| IRM: Drug-loaded nanoparticles (containing MSA-2 and IR).  "W: W" represents weight to weight ratio.  Data represent mean ± standard deviation (n = 3). | | | |


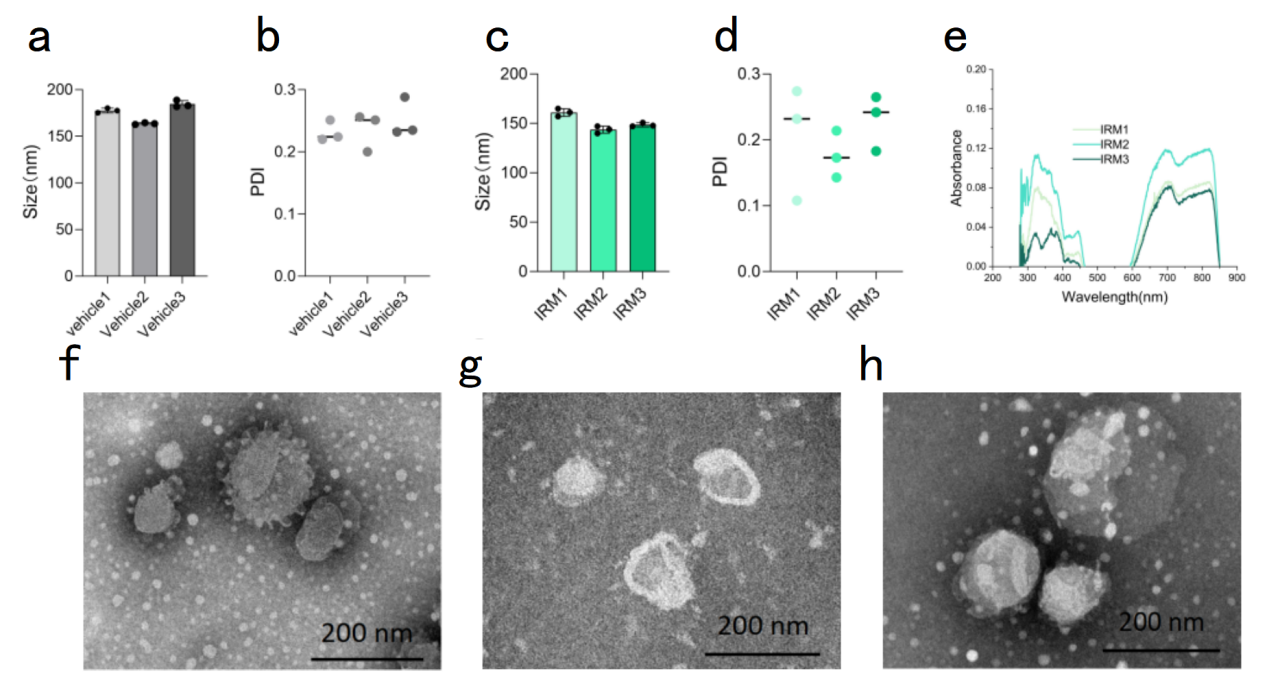


**Figure. S1.** (a, b) Particle size (Size) and polydispersity index (PDI) of blank nanocarriers (Vehicle) with different preparation ratios, as measured by dynamic light scattering (DLS). (c, d) Size and PDI of IRM with different mass ratios of MSA-2 to IR, as measured by DLS. (e) Absorption peaks of IRM nanoparticles with different ratios at 327 nm (characteristic wavelength of MSA-2) and 800 nm (characteristic wavelength of IR), as measured by ultraviolet-visible spectrophotometry. (f-h) Morphology of IRM 1, IRM 2, and IRM 3 nanoparticles as observed by TEM.

| **Table. S3.** Calculation of encapsulation efficiency and drug loading capacity of nanodrugs. | | | | | |
| --- | --- | --- | --- | --- | --- |
| Formulation | Absorbance of MSA-2 | Absorbance of IIR | Concentration (mg) | DLC% | EE% |
| MES | 0.08 |  | 7.03 | 1.53% | 70.30% |
| IRE |  | 0.68 | 24.33 | 5.12% | 97.32% |
| IRM (MSA-2) | 0.09 |  | 7.83 | 1.61% | 78.26% |
| IRM (IR) |  | 0.68 | 24.33 | 5.02% | 97.32% |
| DLC% represents drug loading capacity, DLC% = (W_a_/W_all_) × 100%  EE% represents encapsulation efficiency, EE% = (W_a_/W_Initial_) × 100%  Standard curve of MSA-2: Y = 0.015X + 0.0095, R^2^ = 0.9983.  Standard curve of IR-817: Y = 0.1377X - 0.0151, R^2^ = 0.9999.  MSA-2 Dosage: 10 mg, IR Dosage: 25 mg, SPC dosage: 450 mg, Volume of 20% EtOH: 44 mL. | | | | | |


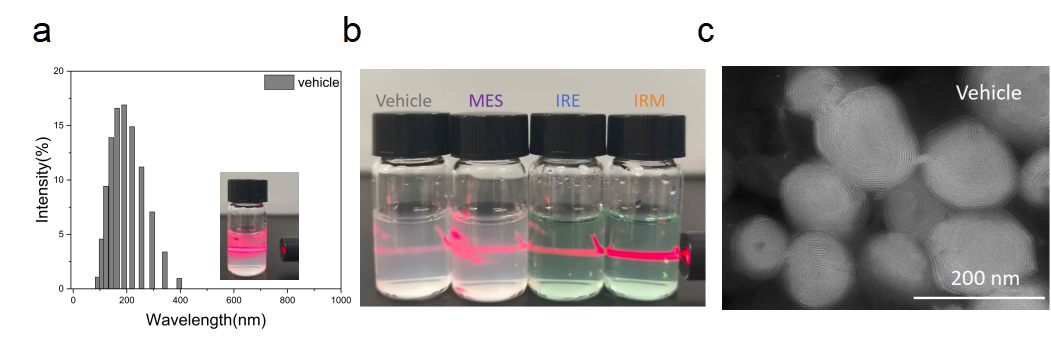


**Figure. S2.** (a) Hydrodynamic sizes of Vehicle measured by DLS and its Tyndall effect. (b) DLS profiles of Vehicle, MES, IRE and IRM. (c) TEM image of the Vehicle.

| **Table. S4.** Size and PDI of MES, IRE and IRM. | | |
| --- | --- | --- |
| Formulation | Size (nm, DLS) | PDI (DLS) |
| MES | 142.43 ± 0.75 | 0.22 ± 0.03 |
| IRE | 132.53 ± 3.31 | 0.25 ± 0.03 |
| IRM | 154.43 ± 1.21 | 0.21 ± 0.03 |
| Data represent mean ± standard deviation (n = 3). | | |


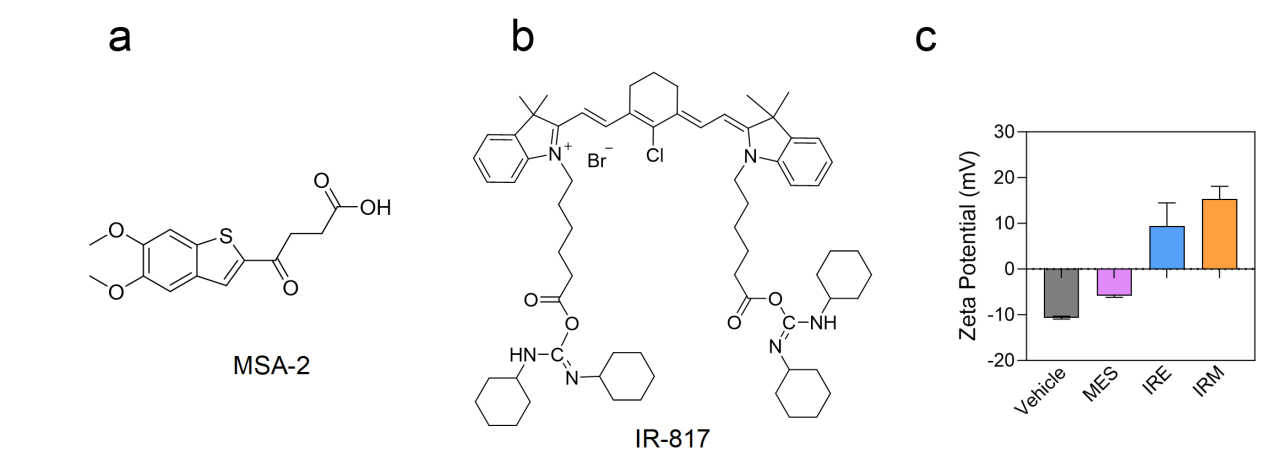


**Figure. S3.** (a, b) Molecular structures of MSA-2 and IR-817 (IR).


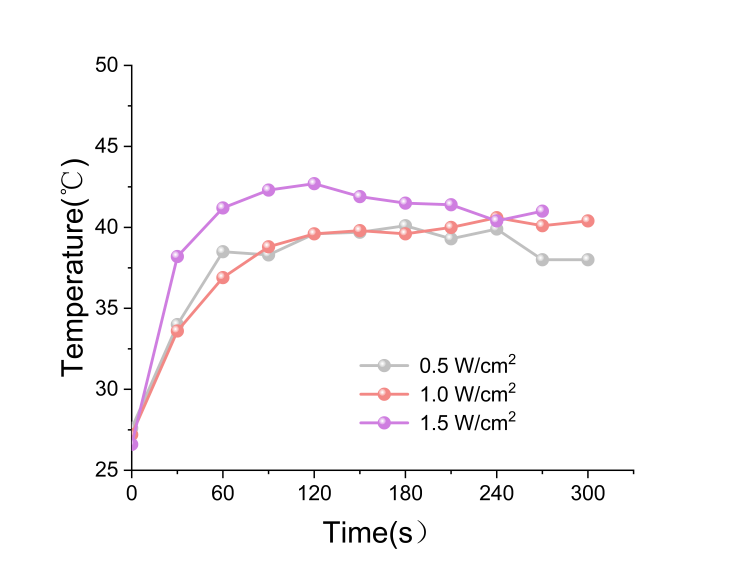


**Figure. S4.** Photothermal heating curves of 30 μg/mL IRM under 808 nm laser irradiation at different laser power densities.


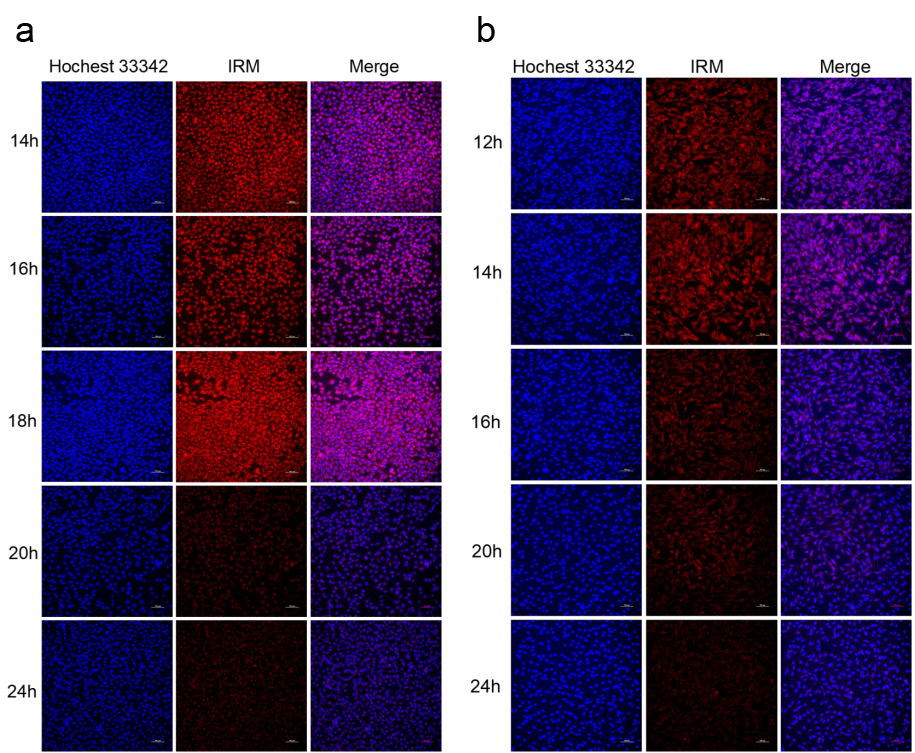


**Figure. S5.** (a) Fluorescence images of A375 cells treated with IRM at different time points (14-24 h). scalebar: 100 μm. (b) Fluorescence images of B16 cells treated with IRM at different time points (14-24 h). scalebar: 100 μm.


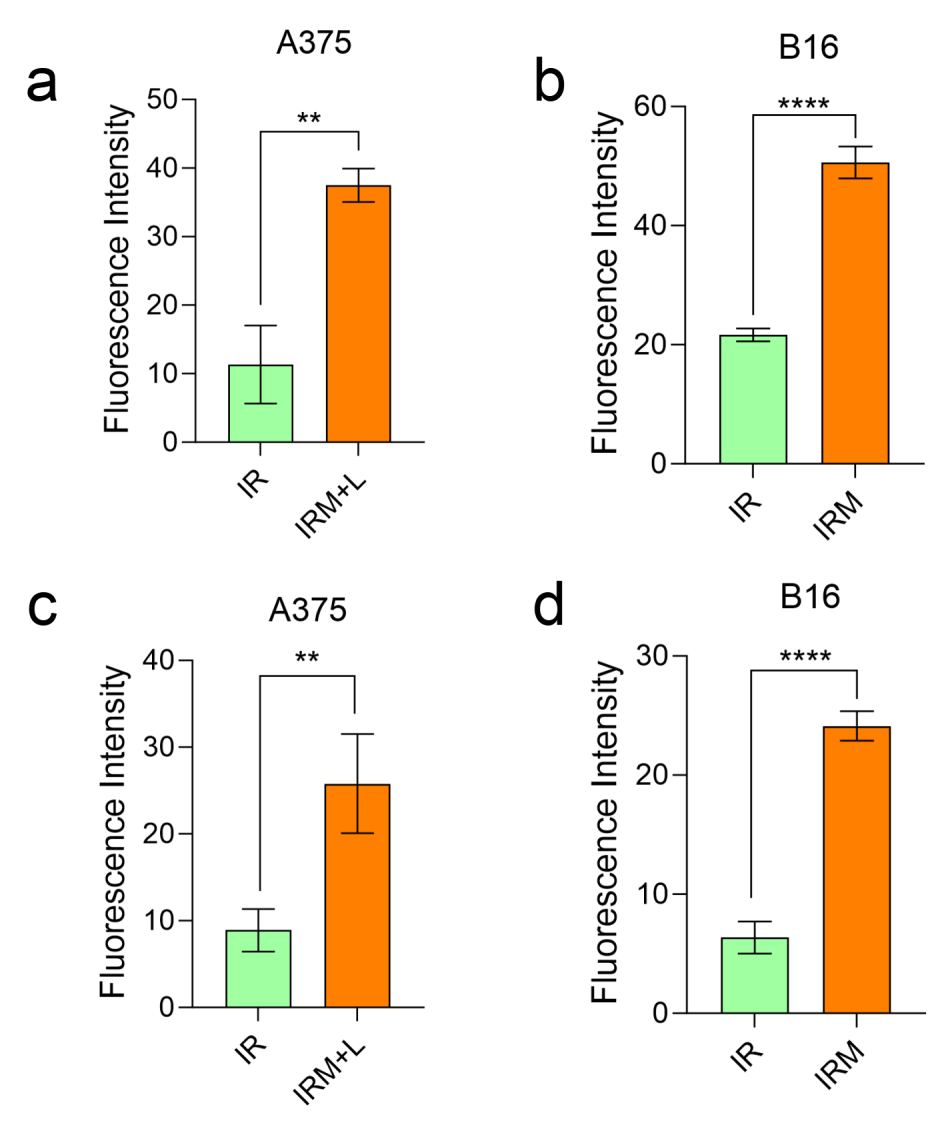


**Figure. S6.** (a, b) Statistical analysis of cellular uptake fluorescence intensity for IR and IRM after 12 h of treatment in A375 and B16 cells, respectively. (c, d) Statistical analysis of cellular uptake fluorescence intensity for IR and IRM after 24 h of treatment in A375 and B16 cells, respectively. Indicates a group which was compared with other groups. All values are the mean ± SD. *P* values, **p* < 0.05, ***p* < 0.01, ****p* < 0.001, *****p* < 0.0001.


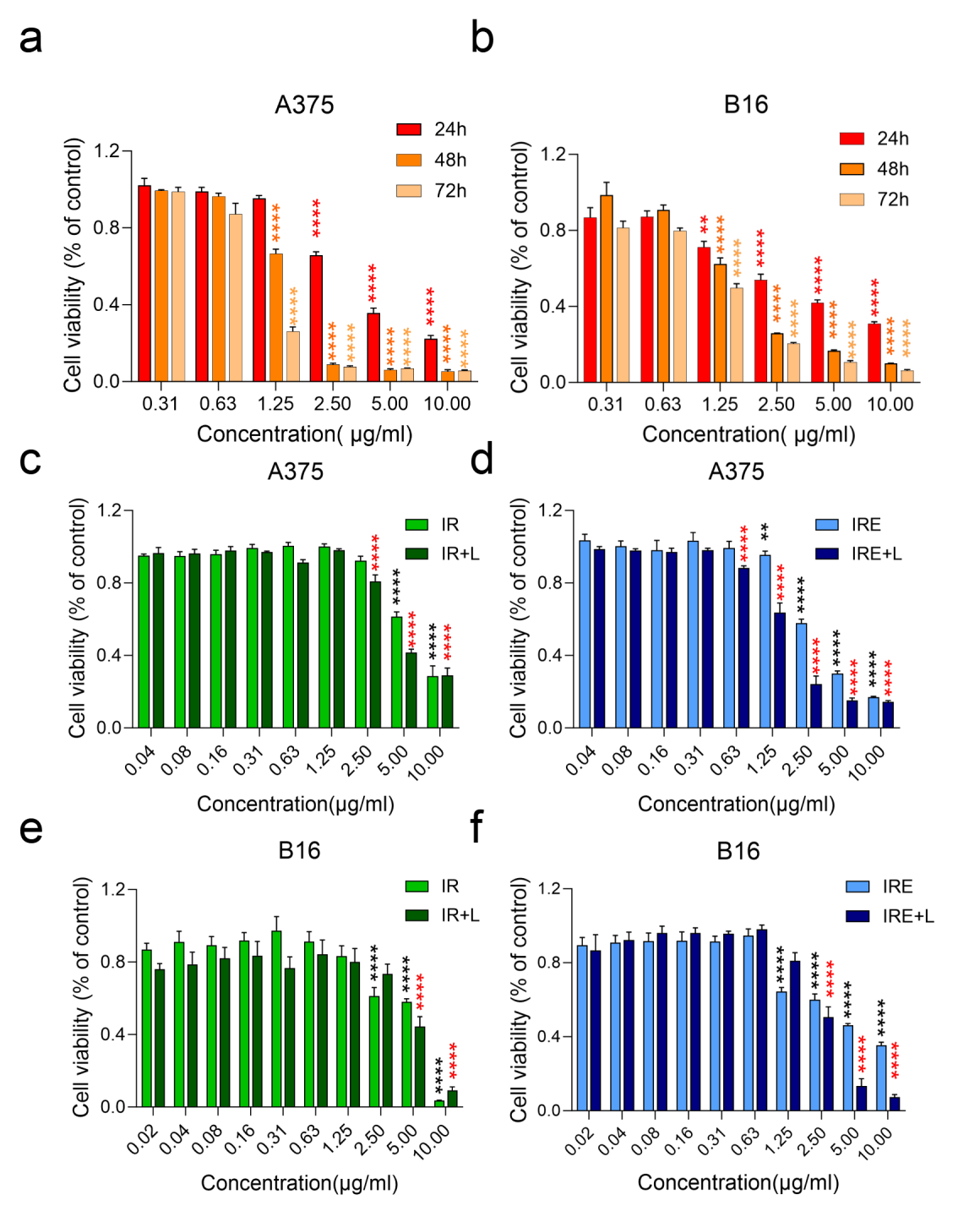


**Figure. S7.** (a,b) MTT assay of A375 and B16 cell viability under different drug concentrations and treatment times (24 h, 48 h and 72 h). (c,e) MTT assay was used to evaluate the cytotoxicity of IR on A375 and B16 cells (with or without laser irradiation) after 24 h of treatment. (d,f) MTT assay was used to evaluate the cytotoxicity of IRE on A375 and B16 cells (with or without laser irradiation) after 24 h of treatment.


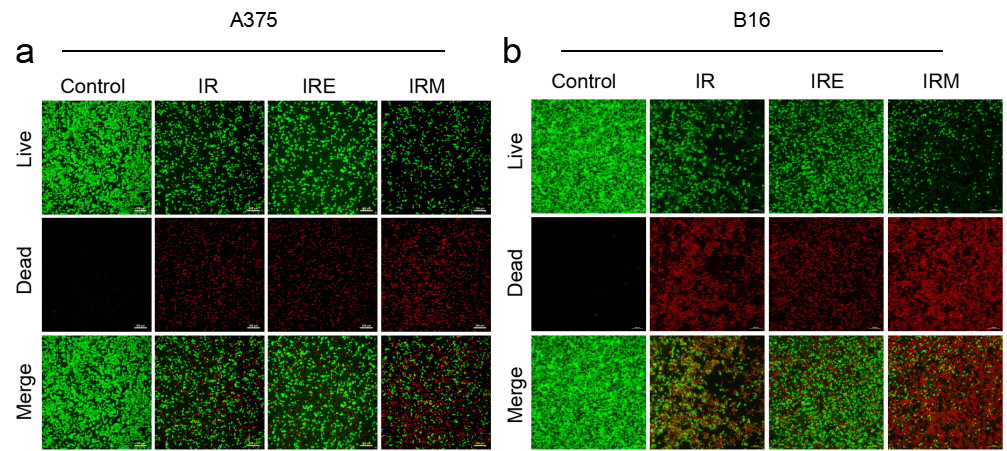


**Figure. S8.** (a) Calcein-AM/PI staining of A375 cells treated with IR, IRE and IRM followed by 808 nm laser irradiation (1.0 W/cm², 5 min), scale bar: 100 μm. Green fluorescence indicates dead cells, while red fluorescence represents live cells. (b) Calcein-AM/PI staining of B16 cells treated with IR, IRE and IRM followed by 808 nm laser irradiation (1.0 W/cm², 5 min). scale bar: 100 μm.


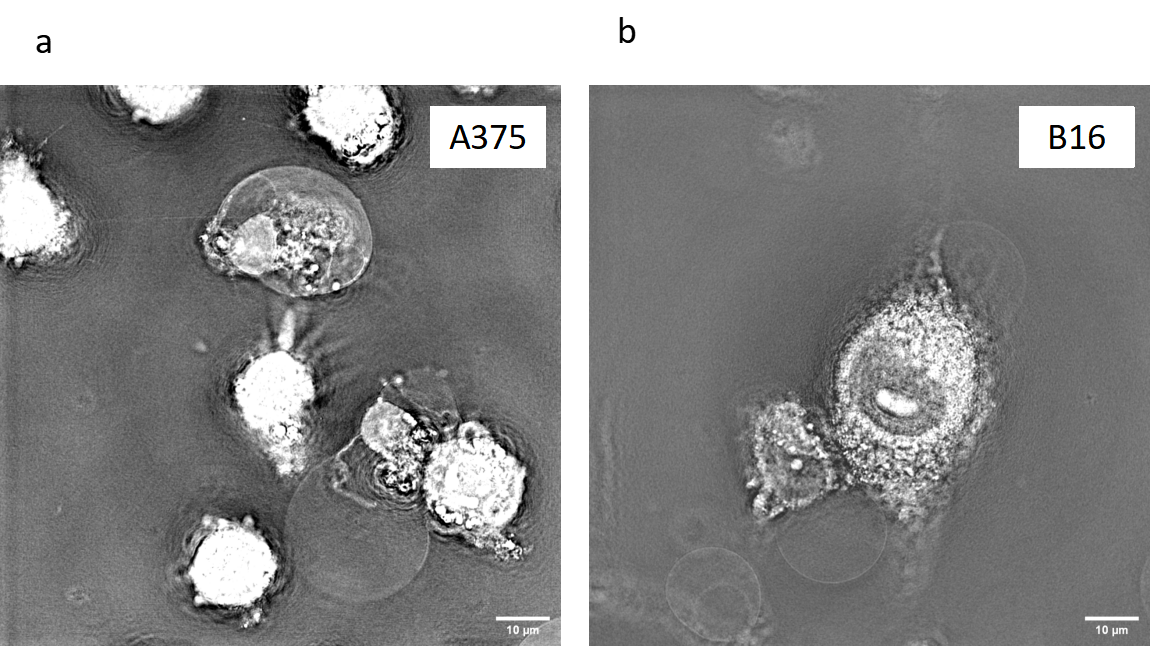


**Figure. S9.** (a,b) Morphology images of pyroptosis and apoptosis in IRM-treated A375 and B16 cells captured using live cell super-reesolution panoramic microscopy, scale bar: 10μm.


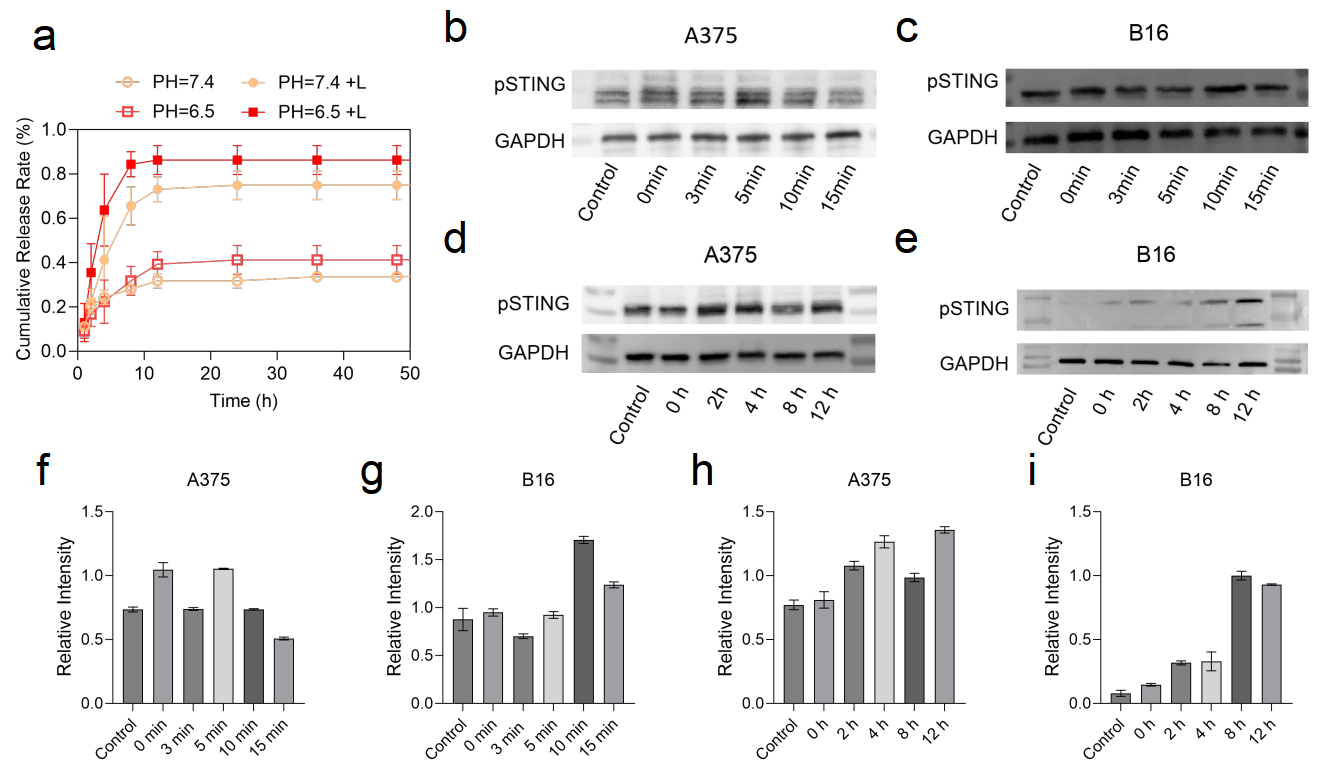


**Figure. S10.** (a) Release of MSA-2 from IRM with or without laser irradiation under different pH conditions. (b, f) pSTING expression in A375 cells treated with IRM under varying laser exposure times (Western blot). (c, g) pSTING expression in B16 cells treated with IRM under varying laser exposure times (Western blot). (d, h) Time-dependent expression of pSTING in A375 cells after IRM treatment and laser irradiation. (e, i) Time-dependent expression of pSTING in B16 cells after IRM treatment and laser irradiation.


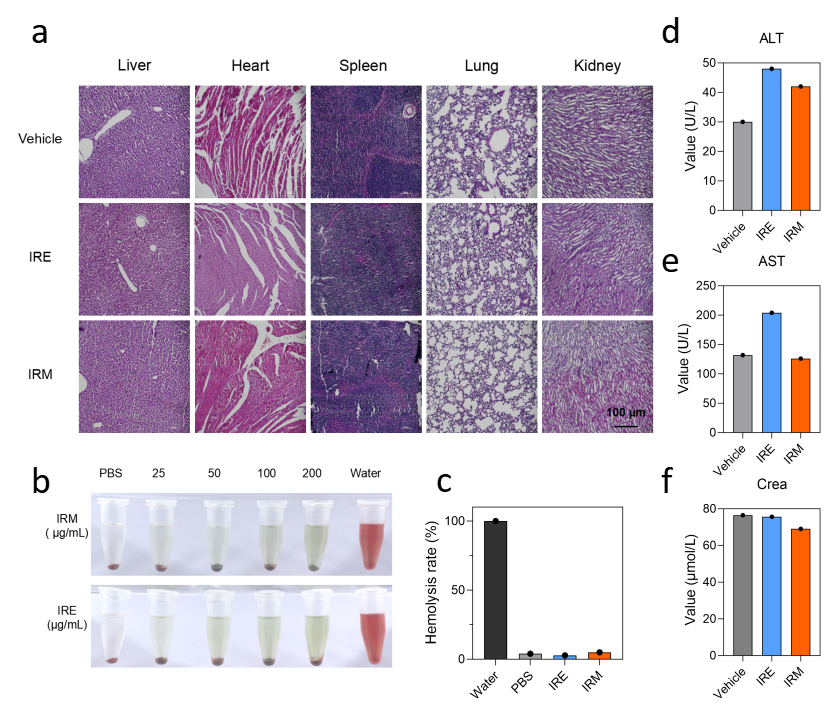


**Figure. S11.** (a) H&E staining of major organs (liver, heart, spleen, lung, and kidney) from mice after different treatments. Scale bar: 100 μm. (b) Photographs taken after the drug was incubated with mouse blood cells. (c) Hemolysis rate after the drug was incubated with mouse blood cells. (d–f) Serum levels of ALT, AST, and Crea in mice after different treatments.


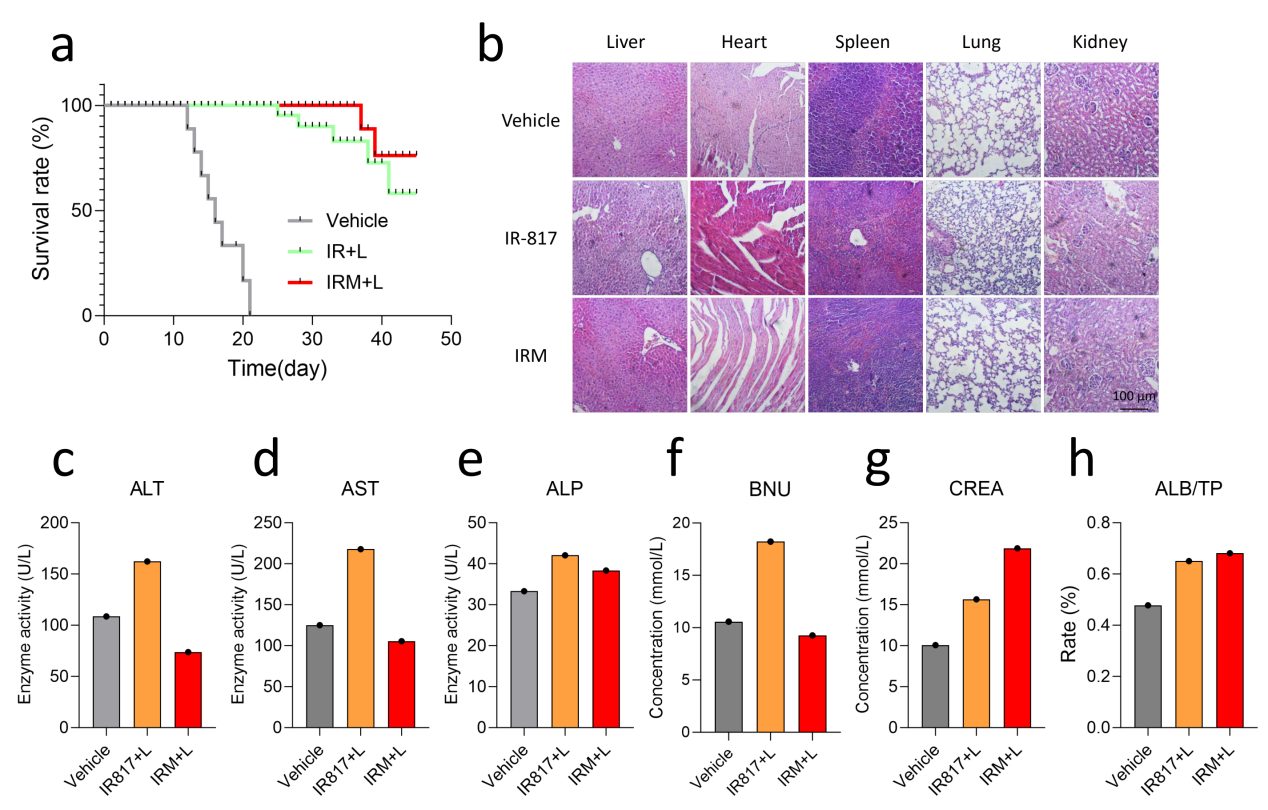


**Figure. S12.** (a) Survival curves of tumor-bearing mice after different treatments during a 45-day observation period. (b) H&E staining of major organs collected from mice at the end of the observation period.(c-e). (c) Alanine transaminase (ALT, unit: U/L). (d) Aspartate transaminase (AST, unit: U/L). (e) Alkaline phosphatase (ALP, unit: U/L). (f) Blood urea nitrogen (BUN, unit: mmol/L). (g) Creatinine (CREA, unit: μmol/L. (h) Albumin/Total protein ratio (ALB/TB). Normal reference ranges of the corresponding indicators in mice: ALT (15–80 U/L), AST (40–160 U/L), ALP (35–135 U/L), BUN (2.0–8.5 mmol/L), CREA (25–85 μmol/L), ALB/TB (0.4–0.6).


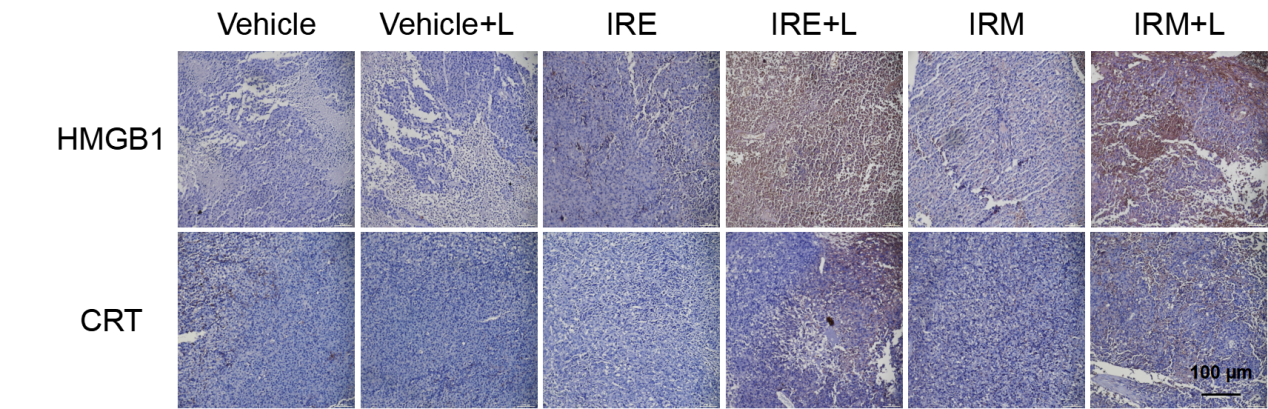


**Figure. S13.** IHC was used to detect changes in the ICD markers (HMGB1 and CRT) in orthotopic tumors.


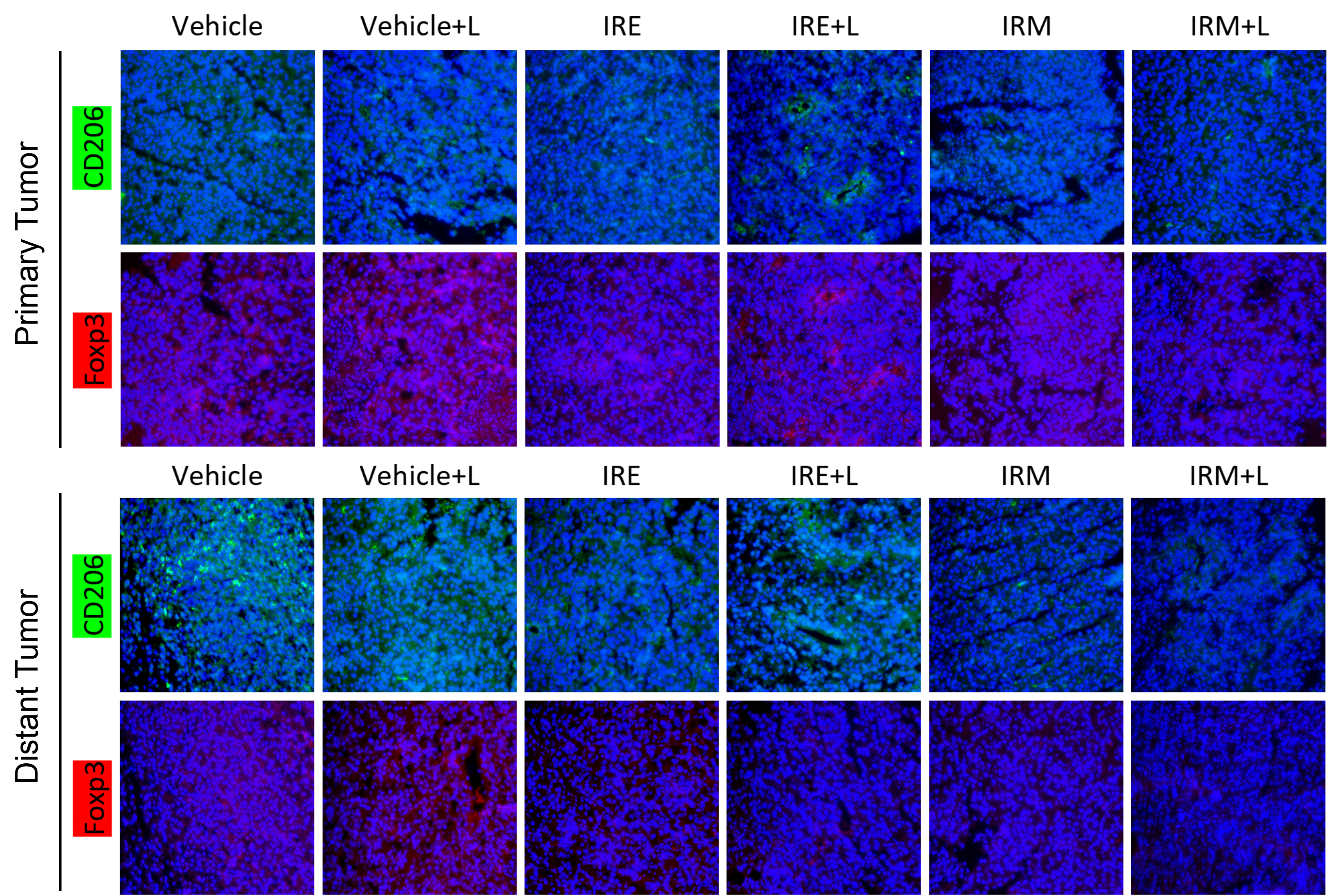


**Figure. S14.** (a) IF analysis of CD206 and Foxp3 expression in Primary mice tumors. (b) IF analysis of CD206 and Foxp3 expression in distant mice tumors.


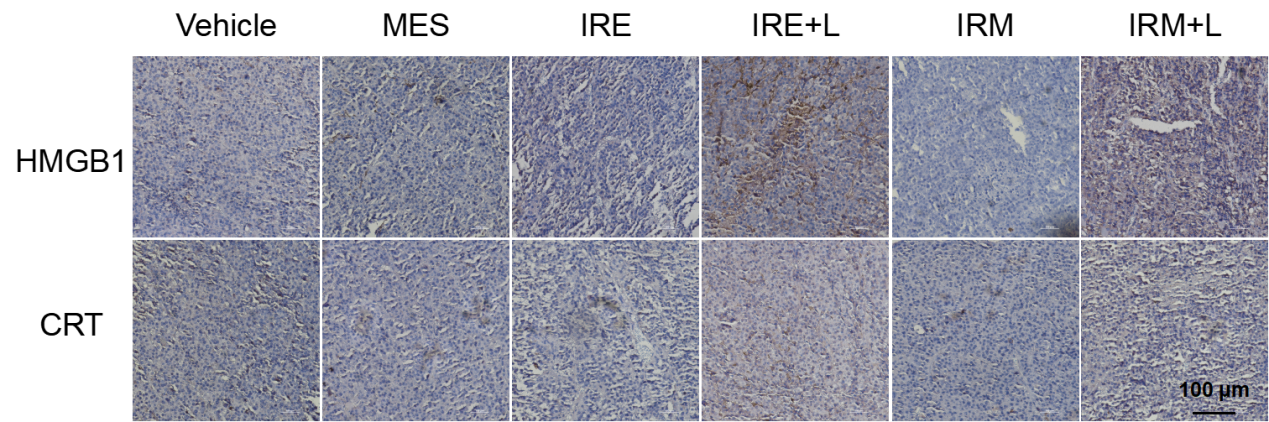


**Figure. S15.** IHC was used to detect the ICD markers (HMGB1 and CRT) in *STING* KO mice primary tumors. scale bar:100 μm.
